# Characterization of Exhausted T Cell Signatures in Pan-Cancer Settings

**DOI:** 10.3390/ijms26052311

**Published:** 2025-03-05

**Authors:** Rifat Tasnim Juthi, Saiful Arefeen Sazed, Manvita Mareboina, Apostolos Zaravinos, Ilias Georgakopoulos-Soares

**Affiliations:** 1 Department of Biochemistry and Molecular Biology, University of Dhaka, Dhaka 1000, Bangladesh; juthirt@gmail.com; 2 Department of Biochemistry and Molecular Biology, The Pennsylvania State University College of Medicine, Hershey, PA 17033, USA; szs7302@psu.edu (S.A.S.); mmareboina@pennstatehealth.psu.edu (M.M.); 3 Department of Life Sciences, School of Sciences, European University Cyprus, 22006, 1516 Nicosia, Cyprus; 4 Cancer Genetics, Genomics and Systems Biology Laboratory, Basic and Translational Cancer Research Center (BTCRC), 22006, 1516 Nicosia, Cyprus

**Keywords:** multi-omics analysis, pan-cancer, T cell exhaustion, exhausted T cell markers, gene expression, mutation landscape, methylation, immune infiltration, drug sensitivity

## Abstract

T cells play diverse roles in cancer immunology, acting as tumor suppressors, cytotoxic effectors, enhancers of cytotoxic T lymphocyte responses and immune suppressors; providing memory and surveillance; modulating the tumor microenvironment (TME); or activating innate immune cells. However, cancer cells can disrupt T cell function, leading to T cell exhaustion and a weakened immune response against the tumor. The expression of exhausted T cell (Tex) markers plays a pivotal role in shaping the immune landscape of multiple cancers. Our aim was to systematically investigate the role of known T cell exhaustion (Tex) markers across multiple cancers while exploring their molecular interactions, mutation profiles, and potential implications for immunotherapy. The mRNA expression profile of six Tex markers, *LAG-3*, *PDCD1*, *TIGIT*, *HAVCR2*, *CXCL13*, and *LAYN* was investigated in pan-cancer. Utilizing data from The Cancer Genome Atlas (TCGA), Gene Expression Omnibus (GEO), The Cancer Proteome Atlas (TCPA), and other repositories, we characterized the differential expression of the Tex markers, their association with the patients’ survival outcome, and their mutation profile in multiple cancers. Additionally, we analyzed the effects on cancer-related pathways and immune infiltration within the TME, offering valuable insights into mechanisms of cancer immune evasion and progression. Finally, the correlation between their expression and sensitivity to multiple anti-cancer drugs was investigated extensively. Differential expression of all six markers was significantly associated with KIRC and poor prognosis in several cancers. They also played a potential activating role in apoptosis, EMT, and hormone ER pathways, as well as a potential inhibitory role in the DNA damage response and RTK oncogenic pathways. Infiltration of different immune cells was also found to be associated with the expression of the Tex-related genes in most cancer types. These findings underline that the reviving of exhausted T cells can be used to enhance the efficacy of immunotherapy in cancer patients.

## 1. Introduction

Cancer remains an enduring global health challenge, prompting extensive research to comprehend its complexities and develop efficacious treatment approaches [1,2]. A key focus in cancer treatment research is understanding the interaction between cancer cells and the immune system. T cells, in particular, play a central role in anti-tumor immunity, with both CD8 and CD4 subsets contributing to immune surveillance and tumor eradication [3,4]. CD8 T cells, often referred to as cytotoxic T lymphocytes (CTLs), are traditionally considered the primary mediators of direct tumor cell killing through the release of perforin and granzymes, as well as through Fas-FasL interactions [5]. However, CD4 T cells are equally critical for effective anti-tumor responses. CD4 T cells support CD8 T cell activation and memory formation by providing cytokines such as IL-2, enhancing dendritic cell function, and sustaining immune cell recruitment to the tumor microenvironment (TME). In certain contexts, CD4 T cells can also exert direct cytotoxic effects on tumor cells expressing MHC class II molecules or facilitate anti-tumor immunity through the secretion of cytokines such as IFN-γ [6]. Additionally, CD4 T cells play a regulatory role in shaping the TME by orchestrating interactions between the innate and adaptive immune systems [7].

Despite the inherent tumor-suppressive capabilities of T cells, tumors can establish suppressive microenvironments that impair T cell function, allowing for tumor persistence and growth [4]. This phenomenon is known as the ’Hellstrom paradox’, suggesting that these initially effective T cells become dysfunctional during tumorigenesis [5].

Furthermore, mutations play a crucial role in driving tumorigenesis, including those leading to T cell dysfunction. T cell dysfunction in cancer is marked by the expression of multiple inhibitory receptors, such as LAG-3 and PD-1, on tumor-infiltrating lymphocytes (TILs). This dysfunction is associated with a reduction in effector functions, including decreased cytokine production and cytotoxicity [5]. Therefore, investigating the molecular underpinnings of T cell exhaustion and dysfunction, particularly in the context of cancer, is essential for developing precise and effective therapeutic interventions. For example, established tools such as Tumor Immune Dysfunction and Exclusion (TIDE) can computationally predict responses to immune checkpoint blockade (ICB) therapy by modeling T cell dysfunction in tumors with higher levels of CTLs and by assessing T cell exclusion in tumors with low CTL levels [8].

T cell exhaustion is a dysfunctional state of T cells that is often seen with chronic infections and cancer [9,10]. Exhaustion is a result of prolonged exposure to antigens, which results in impaired response of these T cells against infected cells. Chronic infections can lead to the state of continued antigen exposure that eventually induces exhaustion in T cells, which is characterized by high expression levels of inhibitory receptor genes, such as *PDCD1* (PD-1), *LAG*-3, *TIGIT*, *HAVCR2* (TIM-3) and immune-modulating genes, such as *LAYN* (layilin) and *CXCL13*, collectively known as “exhausted T cell (Tex)” markers [10,11]. During cancer progression, T cell responses gradually lose functionality, with interleukin-2 (IL-2) production being among the first effector functions to be impaired [10,12,13]. This is typically followed by diminished production of TNF-a and interferon-gamma (IFN-γ) [12,14]. Even though Tex cells are unable to function as a regular T cell population, many markers, such as *CD43*, *CD69*, *CD62L*, and *CD127* are still expressed at similar levels [14]. Although these exhausted T cells are not depleted, their exhaustion can lead to an ineffective response against disease that leads to cancer progression and increased severity [12]. This has been recognized in adenovirus, polyoma virus, and various other viral infections [14]. While much of our understanding of T cell exhaustion comes from models that utilize viral infections, there is a significant difference between cancer and viral microenvironments [15]. Moreover, the lack of standardized criteria for assessing levels of T cell exhaustion across various tumor types highlights the need for longitudinal studies to better characterize and monitor T cell exhaustion dynamics in cancer [15].

T cell exhaustion can develop due to the activity of a number of transcription factors, and certain T cells can be more prone to exhaustion compared to others [14]. Transcription factors that play a key role in exhaustion include the Nuclear Factor of Activated T cells (NFAT), Interferon Regulatory Factor 4 (IRF4), Basic Leucine Zipper ATF-like Transcription Factor (BATF), T-box expressed in T cells (T-bet), Eomesodermin (EOMES), Nuclear Receptor Subfamily 4 Group A (NR4A), and Thymocyte selection-associated HMG Box (TOX) [16,17]. These factors can influence the overexpression of inhibitory receptors such as PD-1, LAG-3, and TIM-3 on T cells in the TME, thereby dampening T cell activation and prolonging exhaustion [9]. Studying the expression of transcription factors can offer insights into the various states of exhaustion. Despite this heterogeneity in the state of Tex, they collectively affect metabolic activity and present with marked epigenetic rewiring. The effect on metabolic activity can be witnessed in reduced glycolysis and increased fatty acid oxidation [12].

Overall, studying the multifaceted nature of T cell exhaustion and dysfunction is especially important to develop effective therapeutic strategies in the field of cancer immunotherapy. We hypothesized that exhausted T cell markers exhibit distinct molecular and immunological characteristics across different cancer types, influencing immune evasion and therapeutic responses. Through integrative multi-omics analysis, we aimed to systematically characterize these signatures to identify potential biomarkers for immunotherapy. Our findings reveal variations in the expression of these genes in pan-cancer, driven by underlying differences in their mutational landscapes, drug responsiveness, immune cell activity, and clinical outcomes. Our analyses not only validate the expression and pathway involvement of previously reported Tex markers but also uncover novel correlations with tumor immune infiltration and drug sensitivity. Additionally, we highlight the translational relevance of these findings by linking Tex marker expression patterns to potential therapeutic interventions.

## 2. Results

### 2.1. Differential Expression in Pan-Cancer

**Figure 1:**
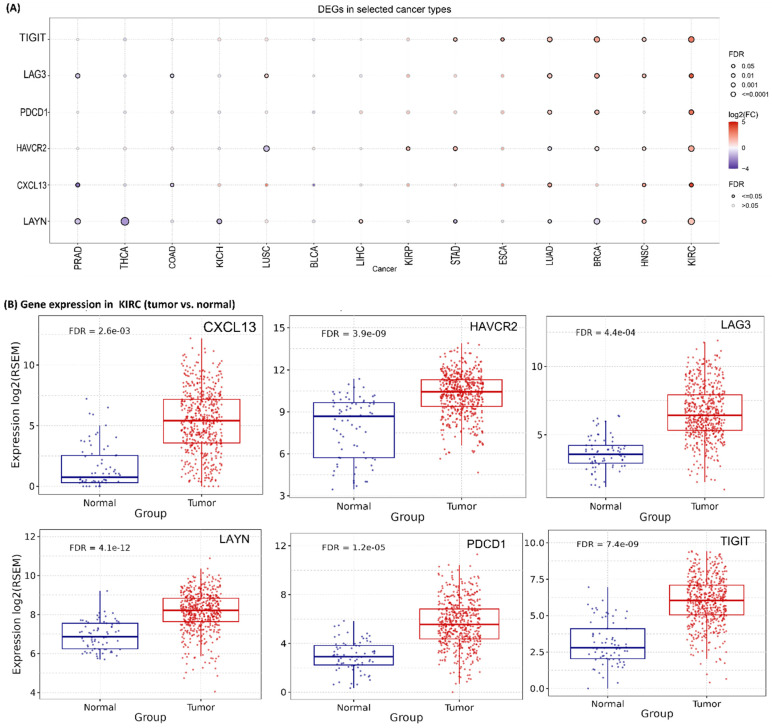

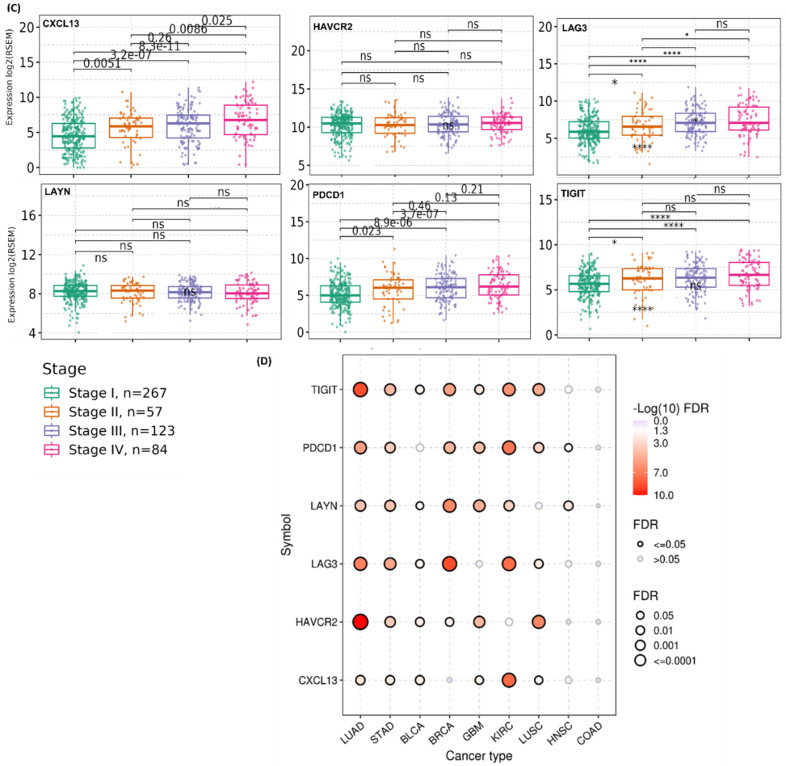
(**A**) Bubble plot illustrating the fold change of six Tex marker genes across 14 cancer types. (**B**) Scattered boxplots showing differential expression of Tex mRNA expression in kidney tumors (KIRC) compared to normal kidney tissues. (**C**) The boxplots summarize the trend of the Tex mRNA expression from early to late stage KIRC. * *p* < 0.05, **** *p* < 0.0001, ns—not significant. (**D**) The bubble plots illustrate the difference between high and low mRNA expression of the Tex marker genes in different cancer types.

The expression of six Tex marker genes was initially investigated across pan-cancer types, revealing higher mRNA levels of *CXCL13* in LUAD, HNSC, and KIRC; *HAVCR2* in KIRP, STAD, and KIRC; *LAG3* in LUAD, BRCA, HNSC, and KIRC; *LAYN* in LIHC, HNSC, and KIRC; *PDCD1* in LUAD, BRCA, and KIRC; and *TIGIT* in STAD, ESCA, LUAD, BRCA, HNSC and KIRC compared to their corresponding normal tissues (Figure 1A). Collectively, all genes exhibited higher expression in KIRC (Figure 1B). In contrast, in PRAD, THCA, COAD, and BLCA tumors, their mRNA levels were significantly lower.

Another point of attention is that the Tex genes that we studied are expressed by both exhausted T cells and activated T cells. To claim that they are exhaustion marker genes in the pan-cancer setting, we evaluated the expression patterns of canonical T cell exhaustion (Tex) marker genes, including *LAG3*, *PDCD1*, *TIGIT*, *HAVCR2*, *CXCL13* and *LAYN*, alongside T cell effector function markers, such as *IFNG*, *GZMB* and *PRF1* in pan-cancer, using TCGA data. All gene expression data were retrieved from the UCSC Xena browser (https://xenabrowser.net/). Our results demonstrate that Tex markers are consistently expressed at varying levels, while the effector genes (*IFNG*, *GZMB*, and *PRF1*) show lower expression levels across most cancer types (Figure S2). This observation aligns with the established understanding that exhausted T cells downregulate effector function genes.

These findings were also corroborated across individual cancer types, where the expression of exhaustion markers was decoupled from that of effector markers, highlighting the distinct transcriptional state of Tex cells. It appears that while activated T cells can transiently upregulate exhaustion markers, the sustained and concurrent expression of multiple Tex genes (e.g., *PDCD1*, *LAG3*, and *TIGIT*) alongside reduced effector gene expression supports their role as indicators of exhaustion rather than activation (Figure S3).

Furthermore, anti-tumor stem-like CD4 and CD8 T cells (also exhaustion precursors) can self-renew and give rise to terminally exhausted T cells as well as effectors, and TCF1^hi^ stem-like T cells, but not terminally exhausted T cells, can be reinvigorated by checkpoint blockades to combat cancers [18]. To understand the dynamics of exhausted T cells, we examined the relationship between the expression of the stem-like markers Tcf7, Slamf6, Lef1, and Bach2 [6,19,20,21] and the exhaustion markers across the pan-cancer dataset. Our results revealed distinct patterns that vary between tumor types but globally resemble those of the Tex markers, and in some cases, they are expressed even higher than some Tex cell markers, like *LAG3*, *PD1*, *TIGIT*, and *CXCL13* (Figure S4). This observation aligns with the established understanding that exhausted T cells downregulate effector function genes. It also reflects that stem-like T cell marker gene expression correlates with exhausted cell gene expression, and these markers are linked to cancer prognosis.

It was further found that *TIGIT*, *LAG3*, *PDCD1*, and *CXCL13* expression was strongly associated with pathological stages in THCA and KIRC and weakly correlated with SKCM, whereas *HAVCR2* and *LAYN* expression displayed the opposite pattern in the majority of the tumors (Figure 1C and Figure S5 and Table S2). Trend analysis also unveiled the differences in Tex genes’ mRNA expression between the pathologic stages within specific cancers. All six Tex genes had a rising trend in their expression from stage I to IV, with ESCA being an example of this (Figure S5 and Table S2). This possibly reflects enhanced immune evasion, as tumors in later stages often upregulate inhibitory checkpoints to escape immune surveillance. Additionally, it was found that the Tex marker gene set expression was poorly related with the subtypes in COAD and HNSC, and, alternatively, highly correlated in LUAD, BRCA, STAD, LUSC, GBM, KIRC, and BLCA (Figure 1D and Table S2).

In summary, the analysis revealed that all six Tex marker genes are associated with differential mRNA expression across different types of cancer, as well as various pathological stages and subtypes, particularly in KIRC.

### 2.2. Survival Outcome in Pan-Cancer

**Figure 2:**
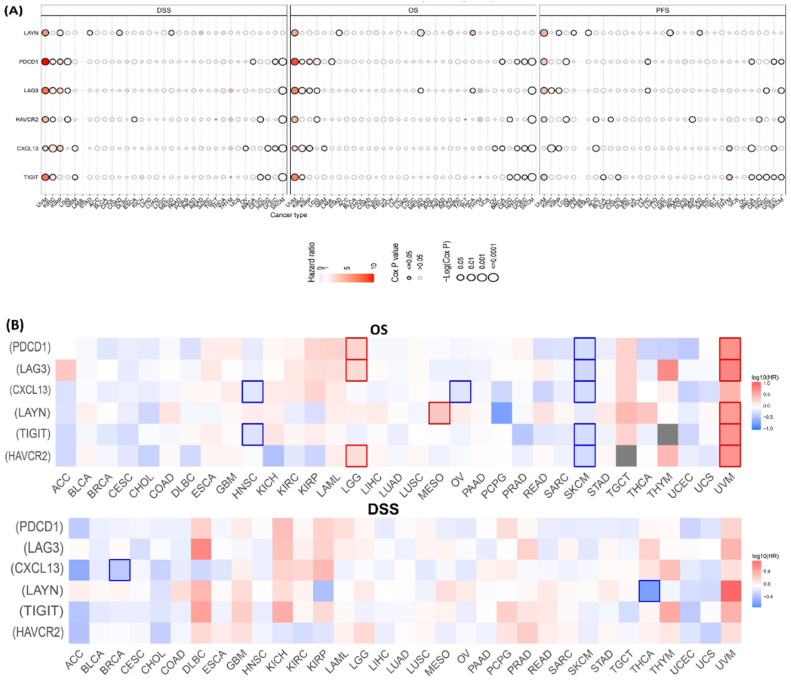

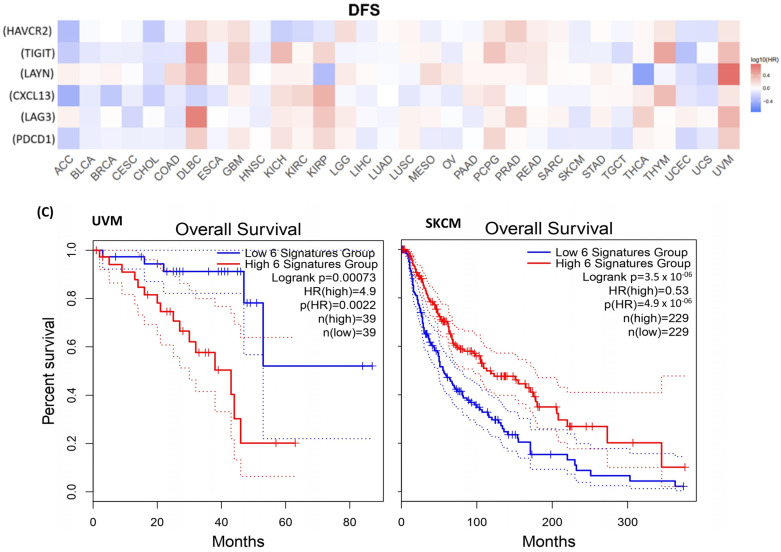
(**A**) Survival outcome difference between the high and low expression group of the Tex marker genes. (**B**) Survival contribution (OS, DSS, and DFS) map of hazard ratio (HR) of the Tex marker genes in pan-cancer. Estimation was conducted using the Mantel–Cox test. Red block, higher risk; blue block, lower risk; darkened outline, significant prognosis. (**C**) Kaplan–Meier overall survival (OS) plots for high and low expression signatures of the Tex marker genes in uveal melanoma (UVM) and skin melanoma (SKCM). Red and blue dotted line represent 95% confidence interval (CI) for each group.

The prognostic value of Tex marker genes was screened by analyzing differences in survival outcomes between high and low Tex mRNA expression groups, which revealed the significant (*p* < 0.05) correlation with survival in various tumor types. The results suggest that the group expressing high levels of Tex mRNA was associated with the worst survival in UVM patients (all six markers with OS and DSS; *LAYN*, *PDCD1*, *LAG3*, *HAVCR2*, and *TIGIT* with PFS) (Figure 2A and Figure S6 and Table S3).

Additionally, GEPIA2 survival analysis was utilized to examine the OS and DSS survival maps of the Tex markers in 33 cancer types. In the case of UVM, the increase in Tex gene expression was related to the poor survival and increased severity of melanoma. Interestingly, in SKCM, the high Tex gene expression resulted in a favorable outcome (Figure 2B and Table S3), suggesting that the role of Tex genes might vary depending on specific genetic or molecular characteristics of the tumor (Figure 2C).

Subsequently, PrognoScan was utilized to construct a univariate Cox proportional hazard regression model to anticipate the prognostic risk of Tex marker genes in pan-cancer and the association between the genes and multiple tumor prognoses (Table S3).

Considering all the pan-cancer prognostic datasets used here, it was found that the expression of six Tex marker genes, either all or at least one, had a significant association with poor prognosis in different cancers. Notably, a disparity between datasets was identified in the case of UVM, which is subjected to further investigation.

### 2.3. Oncogenic Pathway Activity in Pan-Cancer

The exploration of pathway activity differences between high and low mRNA expression of Tex marker genes revealed significant effects on the 10 pathways. It was found that activated *TIGIT* has a potential activating effect on the activity of apoptosis (47%), epithelial to mesenchymal (EMT) (22%), and hormone estrogen receptor (ER) (31%) pathways in pan-cancer. Furthermore, *TIGIT* potentially inhibits DNA damage (16%), hormone androgen receptor (AR) (16%), and receptor tyrosine kinase (RTK) (12%) pathways in pan-cancer.

*PDCD1* expression also revealed a strong activating effect on apoptosis (41%), EMT (22%), hormone AR (12%), hormone ER (28%) and cell cycle (9%) pathways, and a potentially inhibitory effect on hormone AR (6%), hormone ER (9%), RTK (19%) and TSC^mTOR^ (6%) pathways, in pan-cancer. On the DNA damage pathway, *PDCD1* expression was shown to have an equal percentage (12%) of both potentially activating and inhibitory effects.

*LAYN* expression did not reveal much inducing effect on apoptosis (3%) but had a potentially strong activating effect on EMT (38%), hormone ER (16%), RASMAPK (12%), RTK (12%), hormone AR (6%) and TSC^mTOR^ (6%) pathways, in pan-cancer. Alternatively, it strongly inhibited apoptosis (25%) and cell cycle (28%) pathways, along with mild inhibition of DNA damage (19%), hormone AR (12%), hormone ER (9%), RTK (9%) and poor inhibition of TSC^mTOR^ (3%) pathways.

*LAG3* expression, among the six Tex marker genes, activated the apoptosis (53%) pathway the highest. Its high levels were shown to potentially activate the ER (28%), EMT (22%) pathways, the cell cycle (6%), DNA damage (9%), TSC^mTOR^ (6%), and RTK (3%) pathways in pan-cancer. It mostly inhibited the RTK (25%), hormone AR (12%), and DNA damage (9%) pathways along with other pathways to a mild capacity.

The mRNA expression of *HAVCR2* strongly activated apoptosis (31%), EMT (41%), hormone ER (34%), RTK (6%), and PI3KAKT (6%) pathways in pan-cancer. Alternatively, it strongly inhibited hormone AR (25%), RTK and DNA damage (12%), cell cycle (9%), TSC^mTOR^ (9%), and PI3KAKT (9%) pathways.

Lastly, *CXCL13* mRNA expression strongly activated apoptosis (41%), EMT (25%), and hormone AR (9%) pathways. Alternatively, it inhibited hormone AR (12%), hormone ER (12%), DNA damage (9%), PI3KAKT (6%), and RASMAPK (6%) pathways.

**Figure 3:**
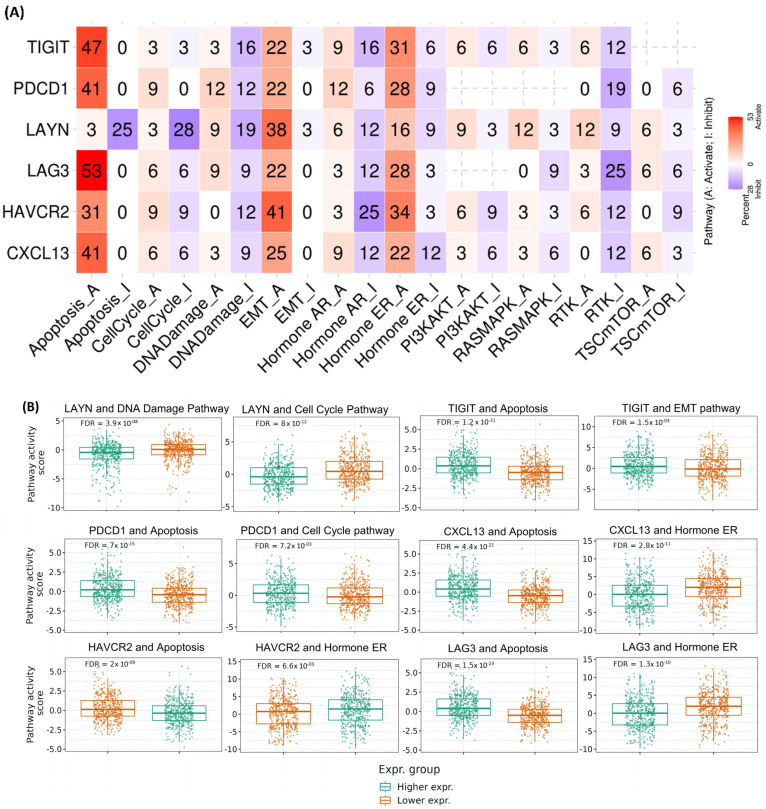
(**A**) Cancers percentage in which the mRNA expression of the six Tex marker genes has a potential effect on the activity of 10 cancer-related pathways. Blue color depicts the shifting of the effect toward inhibition; red color depicts the shifting of the effect toward activation. Each cell contains a percentage (%) representing the proportion of cancer types in which each gene demonstrated a significant association (either inducing or inhibitory) with a specific pathway in pan-cancer. (**B**) PAS of high and low Tex genes’ mRNA expression in breast cancers (BRCA).

In summary, the pathways most induced by Tex mRNA expression in pan-cancer seem to be apoptosis, EMT, hormone AR, hormone ER, DNA damage, and RTK (Figure 3A).

The gene set variation analysis (GSVA) score showed that in the case of UVM, DNA damage, and hormone AR pathways are significantly affected (*p* < 0.05; Spearman’s correlation) by the expression of Tex gene marker set. In the case of pan-kidney cancers (KICH, KIRC, and KIRP), no single pattern was observed for the pathways, whereas lung cancers (LUAD and LUSC) showed almost similar effects. The analysis also showed that the apoptosis pathway was positively affected in 28 types of cancer and negatively affected in UCS, ACC, PCPG, and KICH (Figure S7).

Further, PAS and Tex marker gene expressions were explored. In BRCA, higher PAS values were observed in low *LAYN*-expressing tumors for DNA damage and cell cycle pathways. Higher *PDCD1*-expressing tumors had higher PAS in the cell cycle pathway. The apoptotic pathway was more activated in all high Tex-expressing tumors except *LAYN*. The hormone ER pathway was induced with these Tex expressions, probably due to the relation of this pathway with breast cancer. This pathway had increased activity in low-*HAVCR2*, low-*CXCL13*, and low-*LAG3* expressing tumors. Interestingly, inhibition of this pathway was observed in low-*PDCD1* and low-*TIGIT*-expressing tumors. There was no relationship established with *LAYN*-expressing tumors. The RTK pathway was not induced in these Tex-expressing tumors (Figure 3B and Table S4).

### 2.4. Mutational Profile

#### 2.4.1. SNV Analysis

**Figure 4:**
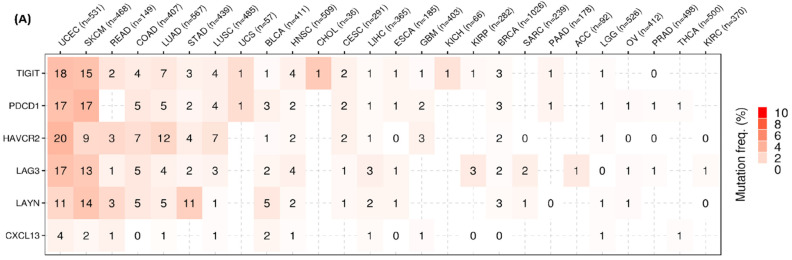

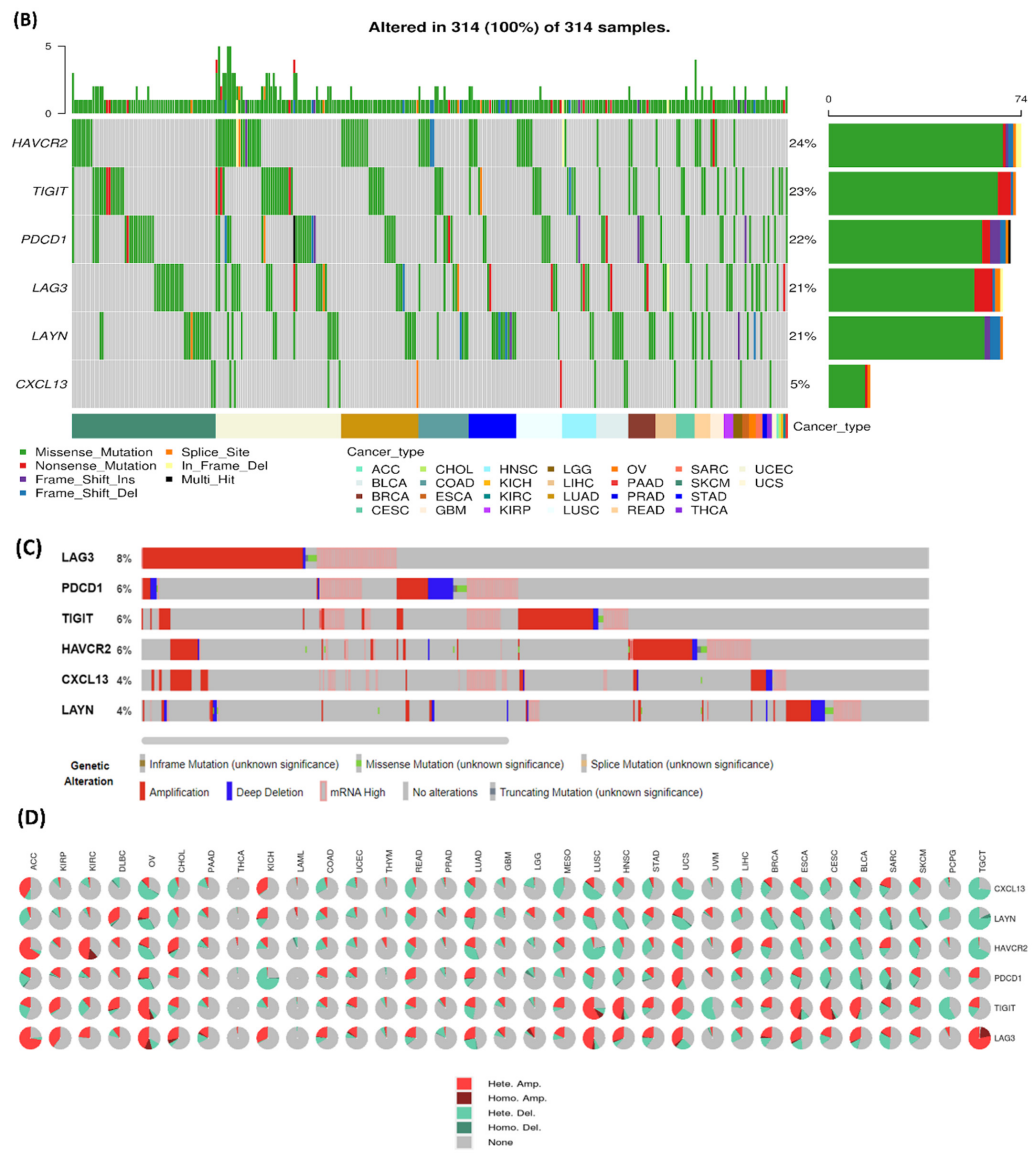

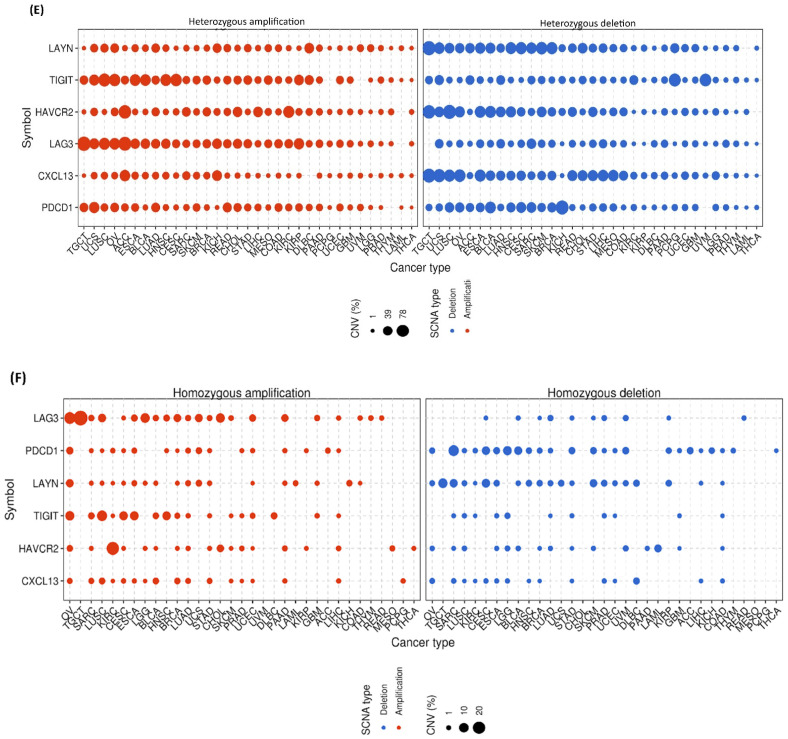

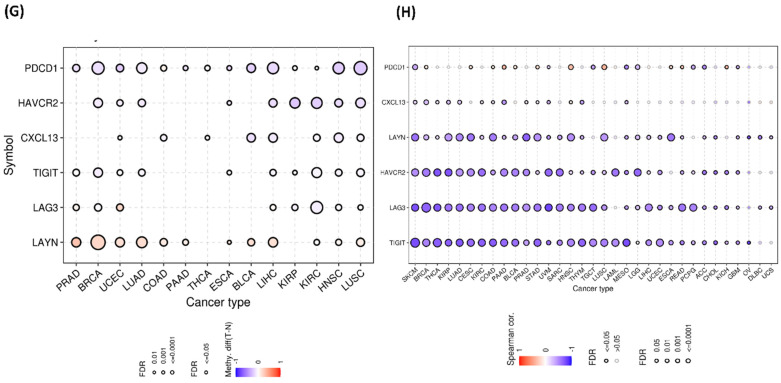
(**A**) Heatmap representing mutation frequency of SNV across pan-cancer. (**B**) Oncoplot representing the frequency of mutation of Tex marker genes in 314 cases and their distribution across selected cancers. Percentage indicates the ratio of genetically altered tumor samples to the total no. of samples. (**C**) Percentage distribution of amplification and deletion of Tex marker genes. (**D**) Pie plot summarizing the CNV of Tex marker genes in the few cancer types. (**E**) Heterozygous CNV profile of Tex marker genes in pan-cancers. (**F**) Homozygous CNV profile of Tex marker genes in pan-cancers. (**G**) Methylation difference of Tex marker genes in selected cancers. (**H**) Methylation and mRNA expression correlation of Tex marker genes in pan-cancers.

The mutation rate of the Tex marker genes was also investigated in pan-cancer. We did not discover any significant differences globally, as the mutation rate was low for all of them. The mutations were found mostly in UCEC and SKCM; 18% of UCEC tumors and 15% of SKCM tumors were mutated in *TIGIT*. The mutation rate for *PDCD1* was 17%, and for *LAG3* it was 17% and 13%, respectively, in both tumor types. The mutation rate that was found to be the highest was for *HAVCR2* (20%) in UCEC. Though *HAVCR2* mutations were identified in 12% of LUAD tumors, other Tex gene mutations were detected in <10% of the LUAD tumors. Mutations in *LAYN* were also found in 14% of SKCM tumors. Last, the *CXCL13* mutation rate was either absent or <5% in all tumors (Figure 4A and Table S5).

Sequencing analysis of the 10,234 samples revealed that every patient within the altered group of 314 carried at least one mutation. Frequent mutations were found for *HAVCR2* (24%), *TIGIT* (23%), *PDCD1* (22%), *LAG3* (21%), *LAYN* (21%) and *CXCL13* (5%) (Figure 4B).

The gene variants mostly contained missense mutations, and the most prevalent type of SNP was cytosine (C) to thymine (T). The whole genome sequencing of the 56 samples also supported these findings. A total of 47 samples had missense mutations, 4 had nonsense mutations, 1 had a frameshift (deletion), 1 had a frameshift (insertion), 1 had an in-frame deletion, 1 had a splice-site mutation, and 1 had a translation start site mutation (Table S5).

The somatic mutation rate of these genes in UCEC ranged from 0.75–3.77%, which is very low. These mutations were scattered across the gene locus, affecting immunoglobulin (Ig) domains, Ig-V set domain, V set domain, chemokine-like domain, and lectin-C type domain (Figure S8). Overall, neither of the six genes was significantly mutated.

Survival outcome analysis of the SNVs for the Tex marker gene suggested that only *LAG3* SNVs affected the survival (DSS and OS) of the HNSC patients (*p* < 0.05 and HR > 0). Interestingly, SNVs of *HAVCR2*, *LAG3*, and *TIGIT* were associated with better survival in UCEC patients (*p* < 0.05, HR < 0 in PFS) (Figure S8 and Table S5).

#### 2.4.2. CNV Analysis

The percentage of CNVs of the Tex markers was assessed to identify the distribution of homozygous and heterozygous mutations across all cancers. The results showed that the heterozygous distribution of amplification and/or deletion mutations of all six genes was present in all 33 cancers. The highest proportion of CNVs was spotted in TGCT, ACC, HNSC, LUSC, CESC, ESCA, KICH, KIRC and OV (>50%). *LAG3* was characterized by a high percentage of heterogeneous amplification, whereas the other five genes had a high percentage of deletions (Figure 4C).

The relationship between each gene’s CNV and its corresponding mRNA expression levels was explored further. No single tumor was found with CNVs in all six genes. However, each gene in different cancers positively correlated (*p* < 0.05) with either its mRNA expression or CNVs, e.g., *TIGIT* in ACC, *LAG3* in UVM, *HAVCR2* in LUSC, *LAYN* in OV and *PDCD1* in BLCA. Interestingly, in KIRP, *CXCL13* CNV was negatively correlated (*p* < 0.05) with mRNA expression (Figure S9 and Table S5).

The effects of the CNVs of Tex markers on the survival outcomes were then investigated. The worst survival outcome (DSS and OS) was seen in UCEC for the CNVs of Tex marker genes. Additionally, *LAG3* CNV was associated with poor survival in PCPG and KICH; *CXCL13* in kidney cancers (KIRC and KIRP) and MESO; *LAYN* in KIRP, *SARC* and LGG (Table S5).

#### 2.4.3. Differential Methylation

The differential methylation of Tex marker genes in 62 tumors and their corresponding normal samples were further analyzed across various cancers. The results indicated that the *LAYN* promoter was hypermethylated in PRAD, BRCA, UCEC, LUAD, BLCA, LIHC, and LUSC tumors. Additionally, the *LAG3* promoter was highly methylated in UCEC, likewise *PDCD1* in COAD and *CXCL13* in THCA. On the other hand, higher methylation of *PDCD1* and *HAVCR2* was found in normal tissues compared to the respective tumors in BRCA, UCEC, kidney tumors, lung tumors, LIHC, and HNSC (Figure 4G and Table S5).

The correlation between differential methylation levels and mRNA expression showed that only *PDCD1* promoter methylation was significantly correlated with its mRNA levels in LUSC, KICH, PAAD, HNSC, READ, ESCA, and STAD. As for *CXCL13*, a positive correlation was detected only in HNSC (Figure 4H and Table S5).

The differential methylation was also noted to have an effect on patient survival outcomes. Methylation levels in the promoter of *HAVCR2* were associated with poor prognosis in kidney cancers (KIRC and KIRP), ACC, UCS, UVM, and LGG. Higher level of methylation of this gene was also correlated with the recurrence of kidney and lung cancers, as well as in ACC, CESC, and CHOL. Alternatively, poor survival and recurrence of kidney cancers, ESCA, and LGG were found to be significantly correlated with the lower methylation in the promoter of *CXCL13*.

Interestingly, in KIRP, poor survival and recurrence were found to be associated with higher methylation of the *LAYN* promoter and lower methylation of *TIGIT* (Figure S10 and Table S5).

In summary, differential methylation of the Tex marker genes posed significant effects on selected cancer types, regarding their expression, and particularly on KIRP patient survival.

### 2.5. Immune Infiltration

#### 2.5.1. mRNA Expression and Immune Infiltration

Tex markers are associated with increased immune infiltration, which can be interpreted in two distinct ways. Elevated immune infiltration may indicate a heightened immune response, where persistent antigen exposure drives T cell exhaustion over time. Alternatively, high Tex expression may play an active role in immune evasion by suppressing T cell function, ultimately weakening the immune response. These contrasting possibilities highlight the complex interplay between Tex markers and immune dynamics in the tumor microenvironment.

**Figure 5:**
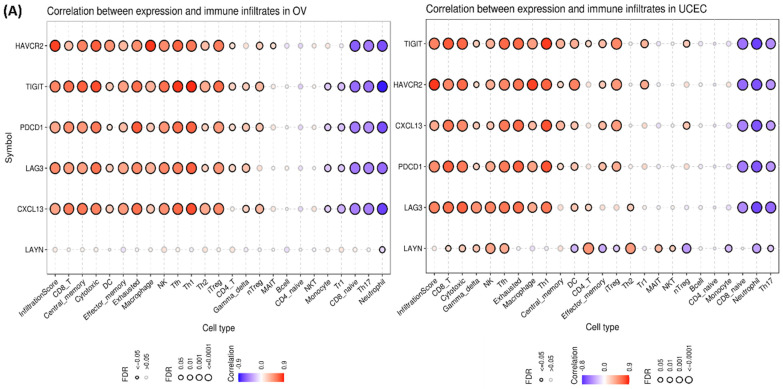

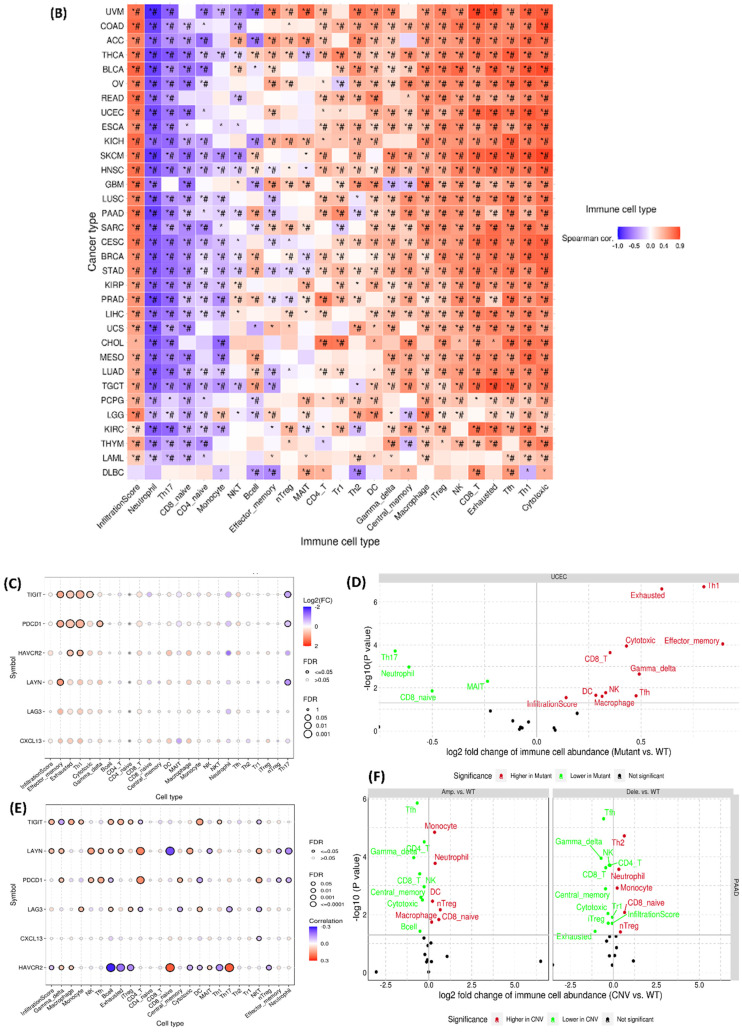

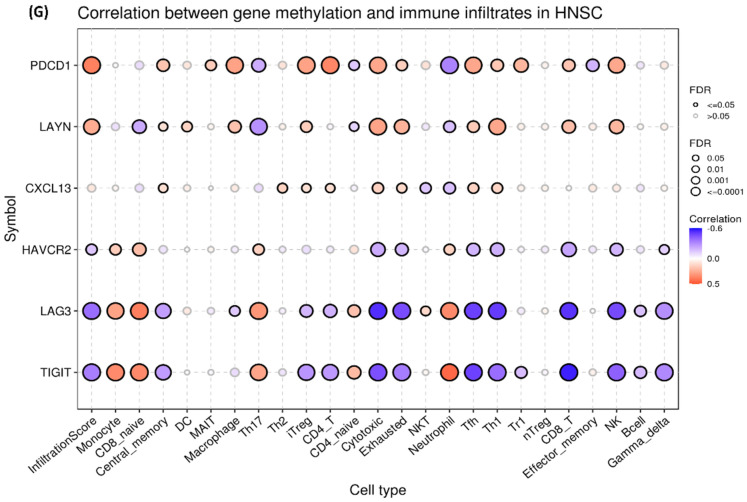
(**A**) Association between Tex mRNA expression and immune infiltrates in OV and UCEC. (**B**) Correlation between the GSVA score and immune cell infiltration in pan-cancer. *: *p* value ≤ 0.05; #: FDR ≤ 0.05. (**C**) Difference of immune cell infiltration between Tex marker WT and mutants in UCEC. (**D**) Disparity of immune cell infiltration between gene set SNV groups in UCEC. (**E**) Correlation between Tex marker CNVs and immune infiltration in BRCA. (**F**) Difference of immune infiltration between gene set CNV groups in PAAD. (**G**) Correlation between methylated Tex markers and immune infiltration in the HNSC.

The correlation between immune infiltrating cells and mRNA expression of Tex markers was further explored. For this analysis, a high immunoscore was considered as an indication of an active immune system and reduced risk of recurrence. Using ImmuneCellAI, we evaluated the 24 immune cell infiltrates and highlighted a significant (positive or negative) correlation between the mRNA expression and infiltration score in OV and UCEC (correlation does not imply causation in this context) (Figure 5A). In OV, all six (but not *LAYN*) mRNA expressions were significantly correlated with the immune infiltrating cells (*p* < 0.05, FDR < 0.0001). In UCEC and a few other cancers, *LAYN* expression showed a variation in its correlation with different immune cells.

It was identified that *HAVCR2* had positive infiltration scores in all cancer types, whereas a negative infiltration score was noted for *CXCL13* (in LGG and DLBCL), *LAG3* (in DLBCL and AML), *LAYN* (in CHOL, KICH, AML, LGG, PCPG, THCA and UCS), *PDCD1* (in DLBCL, AML and THYM) and *TIGIT* (DLBCL and AML). Alternatively, the GSVA score showed a negative correlation with neutrophils and monocytes while demonstrating a positive correlation with CD4 T cells, central memory T cells, cytotoxic T cells, γδ T cells, NK T cells, and Tfh cells (Figure 5B and Table S6). All cancer types were rarely infiltrated with B lymphocytes. The Th1 cells are responsible for activating the neutrophils and macrophages [22], which was found partially true in pan-cancer, where the neutrophil levels were reduced in pan-cancer.

#### 2.5.2. SNVs and Immune Infiltration

The differences in immune infiltrates between WT and Tex mutant tumors were then explored. In UCEC, a significant enrichment of Th1 cells was observed in tumors with *HAVCR2* and *TIGIT* mutations, while effector memory cells were significantly enriched in tumors with *LAYN* and *LAG3* mutations. Significant regression of immune cells in mutant tumors was also found; for example, Th17 cells were infiltrated in *PDCD1* WT cells, while MAIT cells were infiltrated in *CXCL13* WT cells (Figure 5C and Table S7).

The gene set variance of immune infiltrates between SNV mutant and WT tumors was also analyzed in UCEC. In cells with Tex mutations, there was a decrease in the numbers of CD8 naive cells, TH17 cells, MAIT cells, and neutrophils (*p* < 0.05). Conversely, the populations of Th1 cells, Tfh cells, NK cells, macrophages, dendritic cells (DCs), and CD8+ T cells were higher compared to WT cells (Figure 5D and Figure S11).

#### 2.5.3. CNVs and Immune Infiltration

The correlation of immune cells between WT and CNV tumors for Tex genes was further explored. Among 4951 samples, only 217 had a significantly positive correlation (*p* < 0.05, FDR < 0.05) with respect to different immune cells and different cancers. Among them, BRCA showed the highest correlation with infiltration of different immune cells and CNVs in *LAG3*, *PDCD1*, *TIGIT*, *HAVCR2*, and *LAYN*; CNVs in *CXCL13* showed significant anti-correlation with only NKT cells. Alternatively, *LAYN* showed significant correlation with infiltration of CD4 T, NK, cytotoxic, NKT, Tfh, γδ, and B cell; *LAG3* with infiltration of monocytes, DC, iTreg and B cell; *HAVCR2* with CD8 naive cell, Th17, γδ, MAIT and macrophages; *PDCD1* with CD4 T, Tfh, NKT, central memory, NK, and γδ cells; and *TIGIT* with DC, macrophages, B cells, monocytes, iTreg and Th1 cells (Figure 5E and Figure S11 and Table S6).

Significant anti-correlation was also seen between CNVs and immune infiltration. For example, *HAVCR2* CNVs were negatively correlated with infiltration of B cell, iTreg, Th1, nTreg, cytotoxic, and DC; *LAG3* with Th17, NKT, and CD4+ T cell; *LAYN* with CD8 naive cell, neutrophil, MAIT and effector memory cells; *PDCD1* with infiltration of DC, neutrophil, effector memory and B cells; and *TIGIT* with γδ, NKT and CD8 T cells in breast cancer (Table S7 and Figure 5E).

The difference in immune infiltrates between gene set CNV mutant and WT tumors was also analyzed. In PAAD, a significant positive correlation was found for various immune cells. Here, the GSVA of CNVs was significantly correlated with Th2, Tfh, central memory, B cell, monocyte, NK, neutrophil, γδ, CD4 T, and CD8 T cells (Figure 5F and Table S7).

#### 2.5.4. Differential Methylation and Immune Infiltration

The associations between methylation in Tex markers and immune infiltration were further examined in pan-cancer. Six methylated Tex genes were correlated with different immune infiltrates in BRCA, HNSC, KIRP, LGG, LUAD, LUSC, PAAD, PRAD, SARC, SKCM, TGCT, THCA, THYM, and UVM (Table S6). Additionally, it was also identified that all six methylated genes significantly but inversely correlated with different immune cells in BLCA, BRCA, CESC, COAD, ESCA, HNSC, KIRC, KIRP, LGG, LIHC, LUAD, LUSC, PAAD, PRAD, SARC, SKCM, STAD, TGCT, THCA and THYM.

In HNSC, it was found that mostly methylated-*LAYN* and methylated-*PDCD1* showed significantly positive correlation with different immune cells, such as CD8 T, central memory cells, cytotoxic T cells, exhausted T cells, iTeg cells, macrophages, NK, Tfh and Th1 cells. In the case of CD4 naive cells, CD8 naive cells, monocytes, and neutrophils, methylation of *LAG3*, *TIGIT*, and *HAVCR2* showed significant correlation. Methylated-*CXCL13* showed a lower but significant correlation with cytotoxic T cells, Th1, Th2, Tfh, iTreg, CD4 T cells, and central memory cells.

*LAYN* methylation was found to have a positive correlation in KIRP, with the highest number of infiltrated cells being CD8 T cells, Central memory cells, cytotoxic T cells, DC, exhausted T cells, MAIT, Macrophages, NK, NKT, Tfh, Th1, Tr1, iTreg and nTreg cells (Table S6).

In all tumor types, *TIGIT* methylation showed a significant correlation with the infiltration of monocytes and/or neutrophils, with at least one or both cell types being affected (*p* < 0.05). As neutrophil infiltration denotes host inflammation, an important hallmark of cancer, TIGIT methylation might have significance in cancer immunology (Table S6) [23]. Interestingly, none of the methylated Tex genes were positively correlated with the immune infiltrates in UCS.

In short, these findings highlighted that methylated Tex markers might pose a significant effect on all cancer types being correlated with all immune cell infiltration.

### 2.6. Drug Sensitivity Analysis

**Figure 6:**
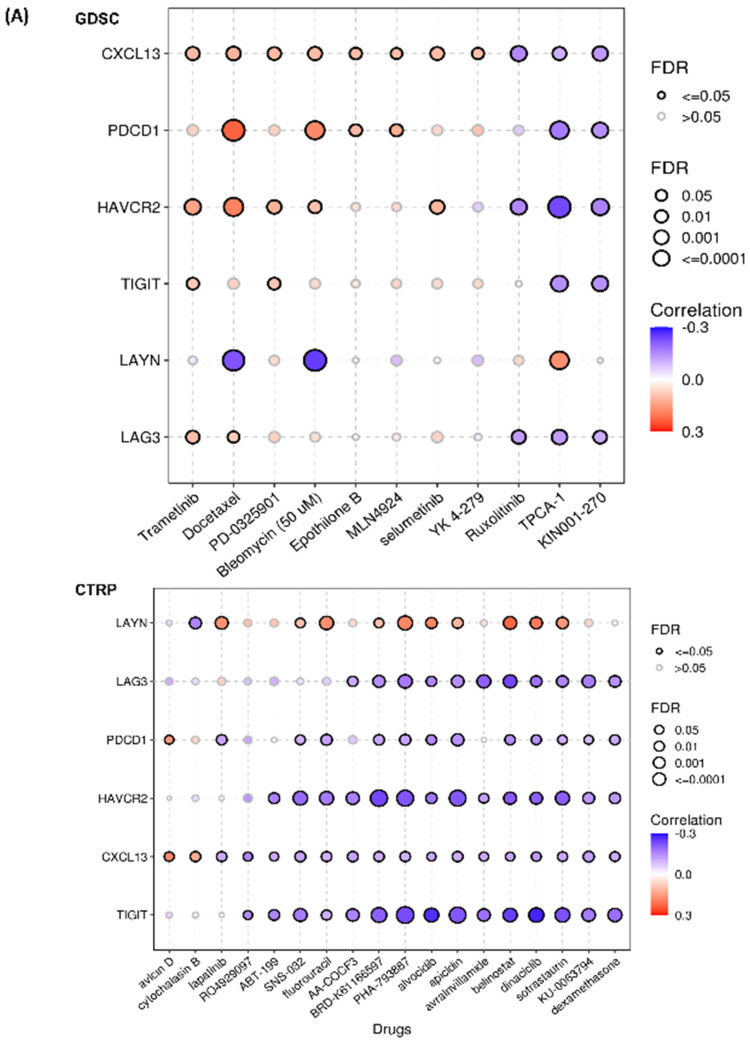

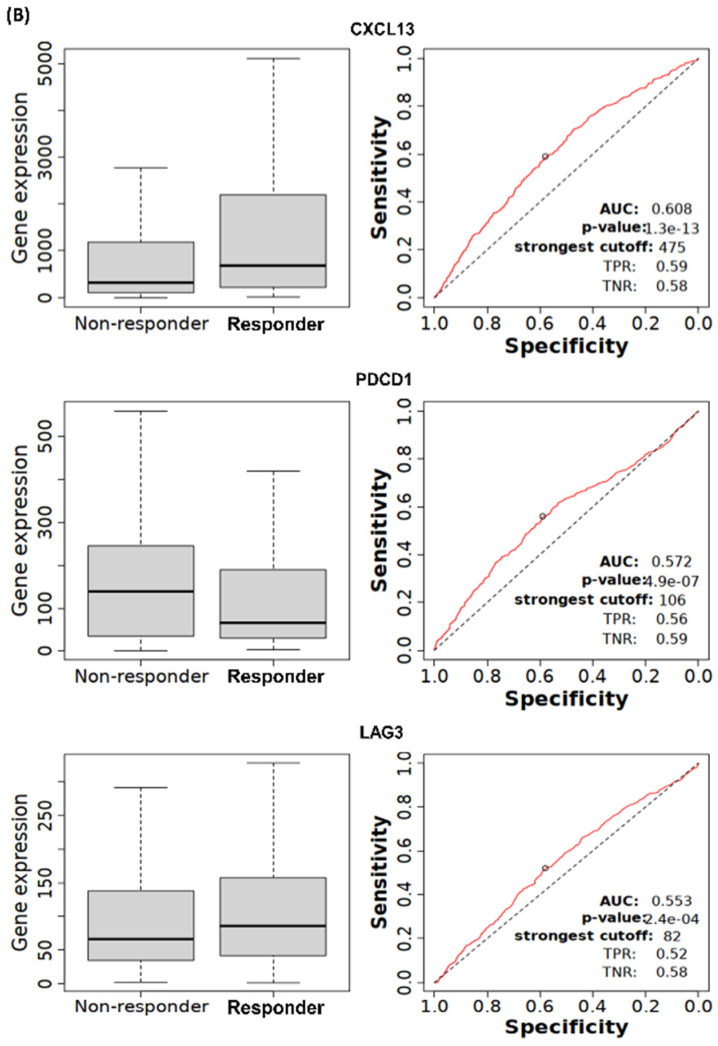

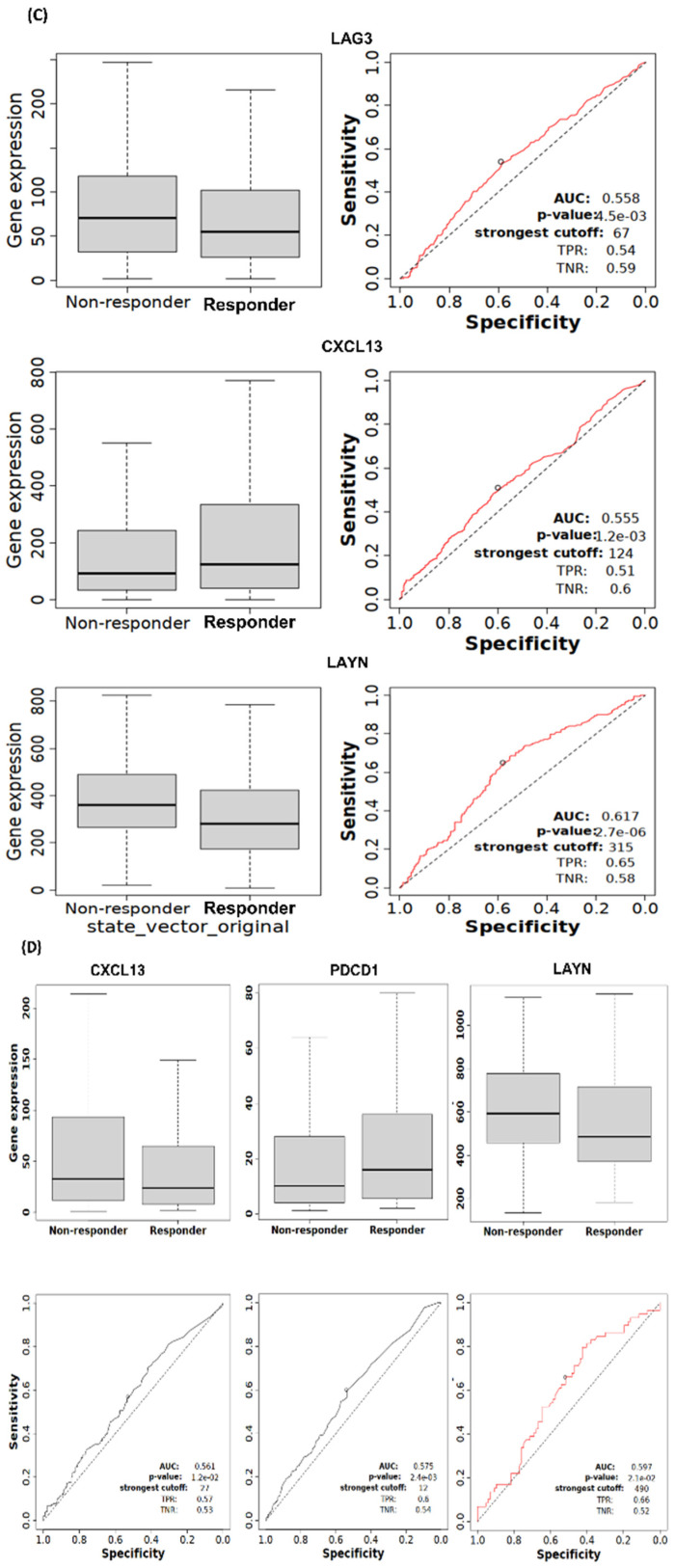

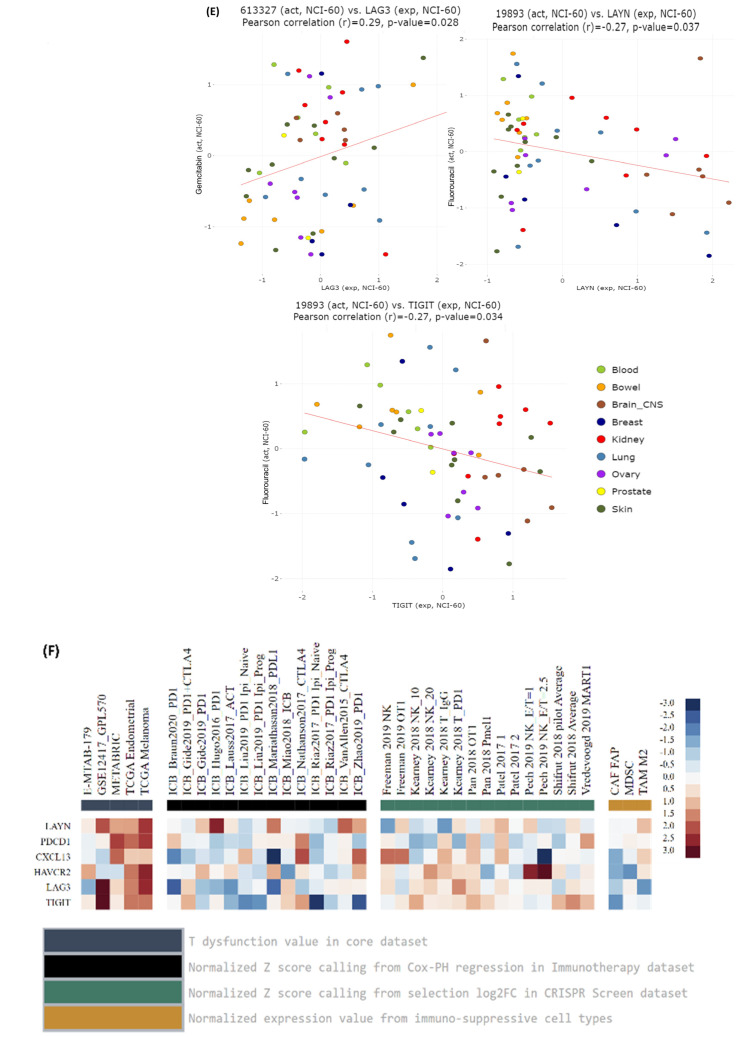
(**A**) Correlation between Tex marker gene expression and IC50 across pan-cancer. (**B**) The ROC plot shows the relationship between Tex mRNA expression and sensitivity in chemotherapy in BRCA. (**C**) The ROC plot showing relationship between Tex mRNA expression and sensitivity in chemotherapy in OV. (**D**) The ROC plot shows the relationship between Tex mRNA expression and sensitivity in chemotherapy in GBM. (**E**) Drug sensitivity analysis of particular Tex marker genes. (**F**) The regulator prioritization clustering heatmap shows the association of Tex with immunosuppression indicators.

For drug sensitivity analysis, the IC50 of multiple drugs covering several cancer cell lines from the GDSC and CTRP databases were retrieved, and their association with the respective Tex marker gene mRNA level was investigated. Interestingly, *CXCL13* mRNA expression was found to be correlated positively with sensitivity in docetaxel in the GDSC database but negatively correlated in CTRP. *CXCL13* also showed positive correlation (resistance) to trametinib, bleomycin, epothilone B, PD-0325901, MLN4924, Selumetinib, YK 4-279, Avicin D and Cytochalasin B, and negative correlation (sensitive) with many other drugs such as Ruxolitinib, TPCA 1, Lapatinib, Fluorouracil, dexamethasone and more. (Figure 6A and Table S7).

In the case of *LAG3*, its expression showed an association with resistance in trametinib, docetaxel, erlotinib, gefitinib, and sensitivity to Vorinostat, 5-Fluorouracil, Navitoclax Methotrexate and more (Table S7). *LAYN* was resistant to 150 compounds in the CTRP database, e.g., lapatinib, 5-Fluorouracil, TPCA-1, alvocidib, apicidin, belinostat, dinaciclib, etc. but showed sensitivity to statin drugs like lovastatin and fluvastatin (Table S7). *HAVCR2* expression was positively correlated with sensitivity and resistance to various drugs. Increased *HAVCR2* expression led to resistance to trametinib, docetaxel, PD-0325901, bleomycin, selumetinib, and austocystin D, while its enhanced sensitivity to crizotinib, methotrexate, 5-fluorouracil, neopeltolide, apicidin, and doxorubicin, among other drugs (Table S7). *PDCD1* was found to be resistant to docetaxel, bleomycin, avicin D, 17-AAG, avicin D, simvastatin and fluvastatin, and sensitive to TPCA-1, flurouracil, apicidin, etc. Likewise, *CXCL13*, *PDCD1* mRNA expression, was resistant to lapatinib in the GDSC database but sensitive in the CTRP database (Figure 6A and Table S7). *TIGIT* showed resistance to trametinib, 17-AAG, VAF-347, and austocystin D but showed sensitivity to alvocidib, belinostat, dinaciclib, and dexamethasone among many other drugs (Figure 6A and Table S7).

The ROC plots showed that the Tex marker genes’ expression levels were significantly associated with the chemotherapeutic response in BRCA patients (*p* < 0.05) (Figure 6B). In the case of anti-Her2 therapy, only *LAYN* showed sensitivity (*p* < 0.05). None of the markers showed any sensitivity to endocrine therapy, but *LAG3* and *HAVCR2* showed significant sensitivity in the case of relapse-free survival at 5 years (Figure S12). In OV, the ROC plot showed a similar relationship with chemotherapeutic response. The pathological response of only *LAG3* was significantly correlated with the chemotherapeutic response in OV patients. In the case of relapse-free responses at 12 months, *CXCL13*, *LAG3*, *LAYN*, and *HAVCR2* showed significant sensitivity to chemotherapy (Figure 6C). In the case of GBM, overall survival data of 16 months in response to chemotherapy was analyzed, and *CXCL13*, *PDCD1*, and *LAYN* were found to be significantly sensitive to chemotherapy (Figure 6D).

Subsequently, the CellMinerCDB was utilized to investigate the correlation between Tex gene expression and sensitivity in a few chemotherapeutic drugs (Figure 6E). Only *LAG3* showed a positive correlation with the sensitivity of gemcitabin. *LAYN* and *TIGIT* showed a significantly negative association with the sensitivity of fluorouracil. The cancer types covered for this analysis were BRCA, kidney tumors, lung tumors, OV, PRAD, SKCM, intestinal cancers, and blood cancers (Figure 6E).

Lastly, the correlation between Tex marker gene expression and patient response to ICB was explored through TIDE. Tex markers were associated with immunosuppressive markers and responses to ICB therapies, such as anti-PD1 and anti-CTLA4, particularly in melanoma, kidney, and lung cancers. These associations extended to measures like T cell dysfunction and exclusion scores and were validated across multiple cohorts through regulator prioritization analyses, which also highlighted findings from CRISPR screens (Figure 6F). There was a varying degree of therapeutic outcome for different therapies and corresponding genes. Higher expression of *LAG3*, *PDCD1*, and *TIGIT* showed the worst survival outcome of anti-ICB therapy in kidney cancer indication drug resistance [24], whereas high *LAG3* and *CXCL13* expressions were associated with better survival of anti-PDL1 therapy in metastatic BLCA [25], indicating drug sensitivity. In the case of anti-PD1 therapy for GBM, there were opposite results for different genes. High *CXCL13* expression was associated with worse survival, but high *LAYN* expression showed improved survival [26]. The anti-PD1 therapy for melanoma offered better survival outcomes when the expression of *PDCD1*, *TIGIT*, and *HAVCR2* was high [27]. Interestingly, high *LAYN* and *HAVCR2* expression levels were found to be associated with anti-PD1 therapy when administered in melanoma and NSCLC-HNSC-melanoma in other studies, respectively (Figure S12C) [28,29].

## 3. Discussion

Recent research on T cell exhaustion suggests that it plays a significant role in the TME and has the potential to be used as a new therapeutic strategy in cancer treatment [8,30]. Therefore, understanding the dynamics and relationship of Tex better across the different cancer types is of key importance in cancer biology and immunology. In this extensive analysis, the detailed correlation of Tex was examined across different cancers. For this purpose, we analyzed the differential expression of six Tex marker genes with respect to the different stages and subtypes of each cancer, survival outcomes, and cancer pathways. Additionally, the mutational profiles of the Tex marker genes and the immune infiltration analysis were systematically investigated for the first time in pan-cancer settings.

In the differential expression analysis, high Tex gene expression was only observed in KIRC, whereas low Tex gene expression was found in PRAD, THCA, COAD, and BLCA. *TIGIT*, *LAG3*, *PDCD1*, and *CXCL13* were highly associated with different stages of cancers, especially in KIRC and THCA. In previous studies, higher expression levels of *LAG3, HAVCR2*, *PDCD1*, *TIGIT*, and *CXCL13* were also found to be upregulated in KIRC [30,31,32,33,34].

Each of the six Tex markers highlights a different aspect of T cell exhaustion—suppressive signaling (PDCD1, LAG-3, TIGIT, HAVCR2), altered chemokine profile (CXCL13), and metabolic or functional adaptation (LAYN). In our study, we explored their co-expression patterns. The reason is that exhausted T cells often co-express multiple inhibitory receptors, and these genes together can identify exhaustion subsets more robustly. In addition, we investigated their therapeutic relevance. As many of these markers are potential or established therapeutic targets, our comprehensive study highlights that they are, indeed, essential for immunotherapy. Therefore, using these Tex markers, we can distinguish truly exhausted T cells from other dysfunctional states, providing insight into their biology and therapeutic potential.

Survival outcomes were also evaluated, and we found that the expression of Tex marker genes was associated with worse survival in UVM and a favorable outcome in SKCM.

In UVM, the poor survival associated with increased Tex gene expression may reflect the immune-evading properties of Tex cells in the immunologically “cold” microenvironment characteristic of this subtype. UVM tumors often lack significant immune infiltration and are typically resistant to ICB therapies. The higher expression of Tex markers in this context may signify advanced T cell exhaustion without sufficient effector activity to mount a productive anti-tumor response. In addition, unlike SKCM, UVM has a lower TMB and a less responsive immune landscape, leading to true exhaustion rather than reversible dysfunction.

In contrast, in SKCM, which is generally considered an immunologically “hot” tumor, increased Tex gene expression may coexist with higher effector activity. This could indicate a dynamic immune microenvironment, where Tex cells still retain partial functionality and contribute to anti-tumor immunity. SKCM also has a high tumor mutational burden (TMB), making it more immunogenic.

Interestingly, *HAVCR2* exhibited protective effects in UVM as per PrognoScan. One of the plausible reasons might be that the Tex genes expression may vary depending on the genetic/molecular profile of an individual. In addition, the AUC values in our model vary between 0.5 and 0.7, which are generally considered indicative of poor performance, suggesting limited clinical applicability of the model. Although this is a limitation in our study, the interpretation depends on the context and application. In our case, the AUC of the ROC curves measure our model’s ability to distinguish between drug responders and non-responders based on gene expression. Collectively, these genes demonstrated poor prognosis for different cancer types and also a protective role for few, which requires further investigation. Previous studies have highlighted the differential expression patterns of *HAVCR2*, *TIGIT*, *LAG3*, *LAYN*, and *CXCL13*, showing strong correlations with survival outcomes across multiple cancer types. These findings underscore their potential role as prognostic biomarkers and therapeutic targets [13,32,33,34,35,36].

The study further explored the activation or inhibition of the 10 cancer-related pathways by the Tex marker genes. Studying these cancer-related pathways is essential for identifying key steps that may serve as effective drug targets. Many of these pathways have also demonstrated sensitivity to various therapeutic drugs [32]. Our study found that the apoptotic pathway was affected by all Tex marker genes except from *LAYN*. Hormone ER and EMT pathways were also activated by Tex marker genes, whereas the RTK pathway was not influenced by their expression at all. The apoptosis, EMT, and hormone ER pathways were previously found to be activated by *CXCL13* [33].

In SNV analysis, there was no significant finding. Only SNVs affecting *LAG3* impacted OS and DSS in HNSC. In UCEC, improved survival was linked to SNVs in *HAVCR2*, *LAG3*, and *TIGIT*. CNV analysis showed both homozygous and heterozygous mutations. While *LAG3* had the highest percentage of heterozygous amplification, the other five Tex genes expressed higher levels of heterozygous deletions. In UCEC, the worst survival was observed for these six Tex genes CNVs.

Although the TCGA database does not provide comprehensive information about normal tissue mutation, we have compared mutation rates in tumors versus matched normal tissues (where available) to determine whether observed mutations in Tex markers are cancer-specific. We observed that both the expression and mutations in T cell exhaustion (Tex) signature genes are infrequent across various normal tissues. This suggests that the expression of Tex markers in the tumor microenvironment is likely influenced more by regulatory mechanisms and external stimuli rather than by inherent genetic mutations.

Exhausted T cell markers in several immune cells within the TME drives the T cell exhaustion indirectly by modulating immune signaling pathways and altering cell-cell interactions. Immune checkpoint ligand for PD-1 and TIM-3 inhibits T cell activation and change cytokine balance to immunosuppressive signals. These ligands are often upregulated on tumor cells, macrophages, and dendritic cells, leading to impaired cytokine signaling and cytotoxic activity in T cells and, finally, T cell exhaustion. Helper T cells and regulatory T cells have been frequently associated with tumor progression and poorer prognoses due to mutations in Tex genes, which can drive immunosuppressive pathways within the TME [37,38]. The relationship between the expression level of *HAVCR2* and cancer immune infiltration, as well as immune checkpoint genes, has been well-documented [13]. *CXCL13* expression levels were found to be associated with these immune infiltrates in this study, which is consistent with a previous study, making it a potential biomarker for different cancer prognosis and immunotherapy outcomes [30]. *LAYN* was also found to be correlated with different immune infiltrates such as macrophages, neutrophils, DC, CD4, and CD8 T cell levels in COAD and STAD, thus contributing to T cell exhaustion [35]. *LAG3* in BRCA [39], *HAVCR2* in GBM, SKCM, UVM, KIRC, CESC [13], and *TIGIT* in SKCM [40] were previously mentioned to be regulating the TME by their association with increased infiltration of immune cells.

The Tex marker genes can be utilized for understanding the TME and its interactions with current treatment therapies. The mRNA expression of these genes showed significant sensitivity and resistance to many chemotherapeutic and anti-cancer drugs. Additionally, the ROC plots indicated significant associations between Tex marker gene expression and chemotherapeutic response in BRCA patients, with LAYN showing sensitivity to anti-HER2 therapy. In OV, *CXCL13*, *LAG3*, *LAYN*, and *HAVCR2* were significantly sensitive to chemotherapy. In GBM, *CXCL13*, *PDCD1*, and *LAYN* were found to be significantly sensitive to chemotherapy. The association between Tex marker gene expression and patient response to ICB therapy was examined through TIDE analysis, revealing varying therapeutic outcomes associated with different therapies and corresponding genes.

Recent research has significantly expanded our understanding of the metabolic and immune-regulatory mechanisms influencing Tex and immune evasion in cancer. It was demonstrated that the hypoxia-inducible factor (HIF) pathway plays a crucial role in tumor progression, immune suppression, and PD-L1 expression, reinforcing the importance of considering tumor hypoxia in Tex-related studies [41]. Another study highlights PD-1 as a tumor suppressor that restricts glycolysis and AP-1 activity in T cell lymphomas, suggesting that metabolic reprogramming contributes to T cell dysfunction and could influence the interpretation of Tex markers across cancers, aligning with the study of HIF-pathway [42]. Similarly, a third study demonstrated that pentose phosphate pathway (PPP) inhibition enhances macrophage-mediated lymphoma clearance, suggesting that targeting metabolic pathways could reprogram the TME to favor immune activation [43]. Metabolic shifts in tumors, particularly those described in the Warburg effect, are well established as modulators of the immune response, and they link tumor metabolism to immune suppression and immune escape [44]. Moreover, it was reported that acidosis within the TME enhances IFN-γ-induced PD-L1 expression in cancer cells, further promoting immune escape [45]. This raises concerns regarding the role of metabolic acidity in shaping immune infiltration patterns observed in the study. Finally, it was also examined how transketolase and vitamin B1 influence ROS-dependent neutrophil extracellular trap formation, which may have implications for immune cell exhaustion in the TME and Tex marker expression [46]. Together, these studies emphasize the necessity of integrating metabolic and immune checkpoint data to refine the current analysis of Tex markers, their prognostic value, and their therapeutic potential in immuno-oncology.

While non-exhausted T cells are generally considered more favorable than exhausted ones, it is more effective in practice to prevent T cell exhaustion from occurring initially. These intricate relationships between Tex marker genes and immune cell behavior provide crucial information about the underlying mechanisms of the cancer response to immunotherapy. This study not only characterizes the exhausted T cell signatures but also provides valuable insights that can be used as a baseline for future research in cancer immunotherapy. There are a few limitations of this study. For example, when analyzing patient survival, age, gender, and tumor stage were not considered, which could impact the observed survival trend. In addition, although we found correlations between Tex markers and drug sensitivity, we need to consider that potential confounding factors, such as co-expressed resistance genes, TMB, or heterogeneity in drug response across different cancer types, could perplex the outcome. Another limitation of our study is the lack of experimental validation. Therefore, this study warrants further research exploring the functional consequences of Tex gene dysregulation and its potential reversibility for the initiation and progression of targeted and personalized anti-cancer therapeutic approaches.

## 4. Materials and Methods

### 4.1. Data Acquisition

We intentionally focused on six specific Tex markers (*LAG-3*, *PDCD1*, *TIGIT*, *HAVCR2*, *CXCL13*, and *LAYN*) due to their significant roles in T cell exhaustion and their known contributions to shaping the TME [2,47,48,49]. To this end, we excluded other markers, such as *CTLA-4*, which primarily regulates T cell priming and activation in lymphoid tissues. In addition, the datasets that we explored did not comprehensively include *CTLA-4*-specific molecular or pathway-level insights that aligned with the study’s pan-cancer objectives. A detailed flowchart describing the workflow of our study is shown in Figure S1.

The data of mRNA expression, copy number variations (CNVs), and methylation of Tex marker genes across 33 cancer types were sourced from the TCGA database through the UCSC Xena browser (https://xenabrowser.net/) (accessed on 15 November 2023) (Table S1). Batch effects were corrected by harmonizing data to minimize technical differences between samples, and mRNA data (RSEM) were normalized prior to their use [50].

### 4.2. Analysis of mRNA Expression

#### 4.2.1. Differential Expression Analysis

We then investigated the variance in Tex expression across 14 cancers from TCGA and their corresponding normal tissues. The sample size for each cancer was as follows: BLCA (*n* = 19), BRCA (*n* = 114), COAD (*n* = 26), ESCA (*n* = 11), HNSC (*n* = 43), KIRC (*n* = 72), KIRP (*n* = 32), KICH (*n* = 25), LIHC (*n* = 50), LUAD (*n* = 58), LUSC (*n* = 51), PRAD (*n* = 52), STAD (*n* = 32), and THCA (*n* = 59). The fold change was determined as the quotient of the average values of the normal and tumor samples. The t test and false discovery rate (FDR) were used to calculate and adjust the *p*-values. FDR ≤ 0.05 was set for statistical significance.

#### 4.2.2. Molecular/Clustering Subtype and Stage Analysis

Subtype-relevant changes in gene expression were explored using clinical data from BRCA, BLCA, COAD, GBM, HNSC, KIRC, LUAD, LUSC, and STAD tumors. The comparison was conducted using the Wilcoxon rank test and ANOVA tests. To analyze the stage, 9478 tumor sample data from all cancer types (except AML, GBM, LGG, PCPG, PRAD, and SARC) cancers were investigated. There were four stages of cancer that we investigated (Clinical, pathologic, International Germ Cell Cancer Collaborative Group, IGCCCG (for tenosynovial giant cell tumors, TGCT, only), and Masaoka (for THYM)). Samples were categorized into stages I, stage II, stage III, and stage IV for pathologic, clinical, and Masaoka stages, while the IGCCCG classified samples into good, intermediate, and poor. The Mann–Kendall trend test was used for trend analysis.

#### 4.2.3. Survival Analysis

In order to determine the interrelationship between mRNA expression and survival outcome, the clinical data of the 33 types of cancer, sourced from the Gene Set Cancer Analysis (GSCA) database [51], were investigated. The prognostic indicators used were overall survival (OS), disease-specific survival (DSS), progression-free survival (PFS), and disease-free survival (DFS). Confounding factors such as age, gender, and tumor stage were considered. Certain samples were excluded from analysis due to deaths unrelated to the specific cancer being studied (for DSS and DFI data). Tumor samples were stratified into high and low expression groups based on the median value, and the survival status was also modeled through the R package survival (version 3.8-0).

Furthermore, GEPIA2 [52] was used to examine patient survival. For this purpose, log-rank tests and Cox proportional hazard models were performed for each of the six genes in every cancer. Differences between low and high mRNA expression were measured by bubble plots and Kaplan–Meier curves.

Additionally, PrognoScan [53] was employed for further survival analysis, using microarray data from Gene Expression Omnibus (GEO) datasets.

#### 4.2.4. Pathway Activity Analysis

The difference between 10 cancer-related pathway activities (activation or inhibition) and gene expression of the Tex markers was estimated and defined by the median pathway score. To assess this pathway activity score (PAS), data of reverse phase protein array (RPPA) for 7876 samples across 32 cancer types were extracted from the TCPA portal (https://www.tcpaportal.org/tcpa, accessed on 15 November 2023). Standard deviation was used to normalize these median-centered data of all samples. Subsequently, the PAS was computed as previously described [54]. In specific, we divided samples into two groups (high and low) by their median gene expression, and the difference in PAS between groups was defined by the student’s t test. The *p*-value was adjusted by FDR, and an FDR ≤ 0.05 was considered significant. When the PAS of a gene with high expression was higher than the PAS of the same gene exhibiting low expression, we considered that this gene might have an activating effect on a pathway; otherwise, it may have an inhibitory effect on it.

The disparity in PAS between low and high expression groups was determined using the student’s t test. Subsequently, the *p*-values were corrected for FDR, with the significant threshold set at 0.05, following the methodologies outlined previously [55,56]. When a sample displayed increased gene expression and simultaneously significantly elevated pathway activity (FDR ≤ 0.05), it was implied that the gene has the potential to stimulate activity on the pathway. Conversely, when the increased gene expression was accompanied by decreased pathway activity, it was considered that the gene had an inhibitory effect on that pathway when overexpressed.

### 4.3. Mutation Profile Analysis

#### 4.3.1. Single Nucleotide Variation (SNV)

The SNV data of 10,234 TCGA samples from 33 cancers were collected, and SNVs of the six Tex marker genes were explored. The dataset contained information about seven types of deleterious mutations, including frameshift insertions or deletions (Indels), in-frame Indels, missense, nonsense, and splice-site mutations. For mutation analysis of the Tex marker genes, whole genome sequencing data of 2922 samples were extracted and, among them, 56 samples had reported mutations [57]. The difference in survival outcome between mutant and wild-type (WT) patients was also assessed using survival in R, Cox proportional hazards model, and the log-rank test. Co-mutation was associated with the clinical outcomes, as described previously [51,58].

#### 4.3.2. Copy Number Variation (CNV)

CNV data from 11,495 patient samples were collected as described above, and GISTIC2.0 was used for the analysis of genomic regions with significant deletions or amplifications [59]. The GISTIC score reflected the CNVs in each gene in the particular tumor. Homozygous or heterozygous amplifications and deletions were presented visually using oncoplots, bubble plots, and pie plots. The correlation between CNVs affecting each of the six Tex genes and their corresponding mRNA expression levels was also analyzed through Spearman’s correlation test, as previously described [60]. To assess the differences in the survival outcomes between CNV-affected and WT groups, samples were divided into WT, deletion, and amplification. For statistical analysis, the survival package (version 3.8-0) and the log-rank test were utilized.

#### 4.3.3. Differential Methylation

The differential methylation patterns between normal and tumor sample groups were analyzed using Illumina HumanMethylation 450 k level 3 data from TCGA. Only cancers containing over 10 pairs of tumor and adjacent normal sample data were used in this analysis. Typically, a single gene region contains multiple methylation sites, each represented by various tags storing their respective methylation levels. For this reason, an association was established to omit the methylation sites that were negatively associated with the gene expression before differential methylation analysis. We assessed the relationship between gene expression and methylation levels using the Spearman correlation test. The *p*-values were FDR-corrected. For the survival analysis regarding methylation data, samples were processed as previously mentioned. The tumor samples were then divided into two groups- high and low methylation levels- and survival difference was measured between these groups.

### 4.4. Immune Infiltration Analysis

To analyze immune infiltration, we extracted information for 10,995 samples from 33 TCGA cancers. These also contained data from 24 immune cells comprising lymphocytes (18 subtypes of T cells, B cells, and natural killer (NK) cells) and myeloid cells (neutrophils, monocytes, macrophages, and dendritic cells (DC)). The estimation of the immune cell abundance was conducted using the Immune Cell Abundance Identifier (http://bioinfo.life.hust.edu.cn/ImmuCellAI/#!/ (accessed on 15 November 2023) [61]. Subsequently, we correlated Tex gene expression with the immune cell infiltration using the Spearman’s test.

The difference in immune cell infiltrates between genes with SNV and WT groups and the correlation between immune cell infiltrates and CNVs of Tex marker genes were estimated using the Wilcoxon rank test. ImmuCellAI was further used to evaluate the infiltrates of the aforementioned immune cells [55,61]. The association between the immune infiltration and the methylation of Tex marker genes was further examined using Spearman’s correlation analysis. FDR was used to adjust *p* value in all of the cases.

### 4.5. Drug Sensitivity and Expression Correlation Analysis

To analyze the anti-cancer drug sensitivity, initially half maximal inhibitory concentration (IC50) data of 265 small molecules in 860 cell lines were retrieved from the Genomics of Drug Sensitivity in Cancer (GDSC, Release 8.4 (July 2022) database [56,62,63]. For the same purpose, another set of data (IC50 of 481 small molecules in 1001 cell lines) was collected from the Genomics of Therapeutics Response Portal (CTRP) [64,65,66]. These data were then merged separately with their corresponding mRNA expression data for correlation analysis (Pearson’s test), offering the association between gene expression and IC50 of a particular drug. The *p*-values were also FDR-adjusted.

CellMiner Cross Database (CDB) was used to investigate the associations between the levels of gene expression and sensitivity to anti-cancer drugs. This database offered the pharmacogenomic data of all cancer cell lines, sourced from GDSC, CTRP, and NCI-DTP NCI-60 (https://discover.nci.nih.gov/cellminercdb/, accessed on 10 January 2024) [67].

The correlation between the Tex mRNA expression and sensitivity in anti-HER2 therapy, endocrine therapy, and chemotherapy in BRCA, as well as chemotherapy in OV and GBM, were explored using the ROC plot (http://www.rocplot.org/ accessed on 15 January 2024). Parameters included pathological complete response, relapse-free survival and OS, and treatment mentioned. The Mann–Whitney test was conducted to compare the Tex marker mRNA expression of responsive and non-responsive groups against the therapies [68].

Furthermore, the TIDE algorithm was further employed to identify the correlation between Tex mRNA expression and ICB therapy outcomes (https://tide.dfci.harvard.edu/ accessed on 15 January 2024) [69,70].

## 5. Conclusions

In conclusion, we explored the differential expression, mutations, and methylation levels of a Tex-specific gene signature in pan-cancer, and we correlated the most interesting results with patient survival, immune infiltration, and pathway activity. Our findings corroborate that the reviving of exhausted T cells can be used to enhance the efficacy of immunotherapy in cancer patients.

## Data Availability

Genomic data were extracted from TCGA (https://portal.gdc.cancer.gov/, accessed on 15 November 2023). Small molecule drugs’ data were extracted from GDSC (https://www.cancerrxgene.org/, accessed on 10 January 2024) and CTRP (https://portals.broadinstitute.org/ctrp/, accessed on 10 January 2024). Immunogenomic data analysis was performed using ImmuCellAI (http://bioinfo.life.hust.edu.cn/ImmuCellAI#!/, accessed on 15 November 2023).

## Supplementary Materials

The following supporting information can be downloaded at: https://www.mdpi.com/article/10.3390/ijms26052311/s1.
