# Supplementary material for: Characterization of Exhausted T Cell Signatures in Pan-Cancer Settings
Source: Int J Mol Sci. 2025 Mar 5;26(5):2311. doi: 10.3390/ijms26052311 (PMC11899893; doi:10.3390/ijms26052311)
Supplement: Supplementary file 1 [file ijms-26-02311-s001.zip › ijms-3441192 Suppl. Figures S1-S12.pdf]

Figure S1

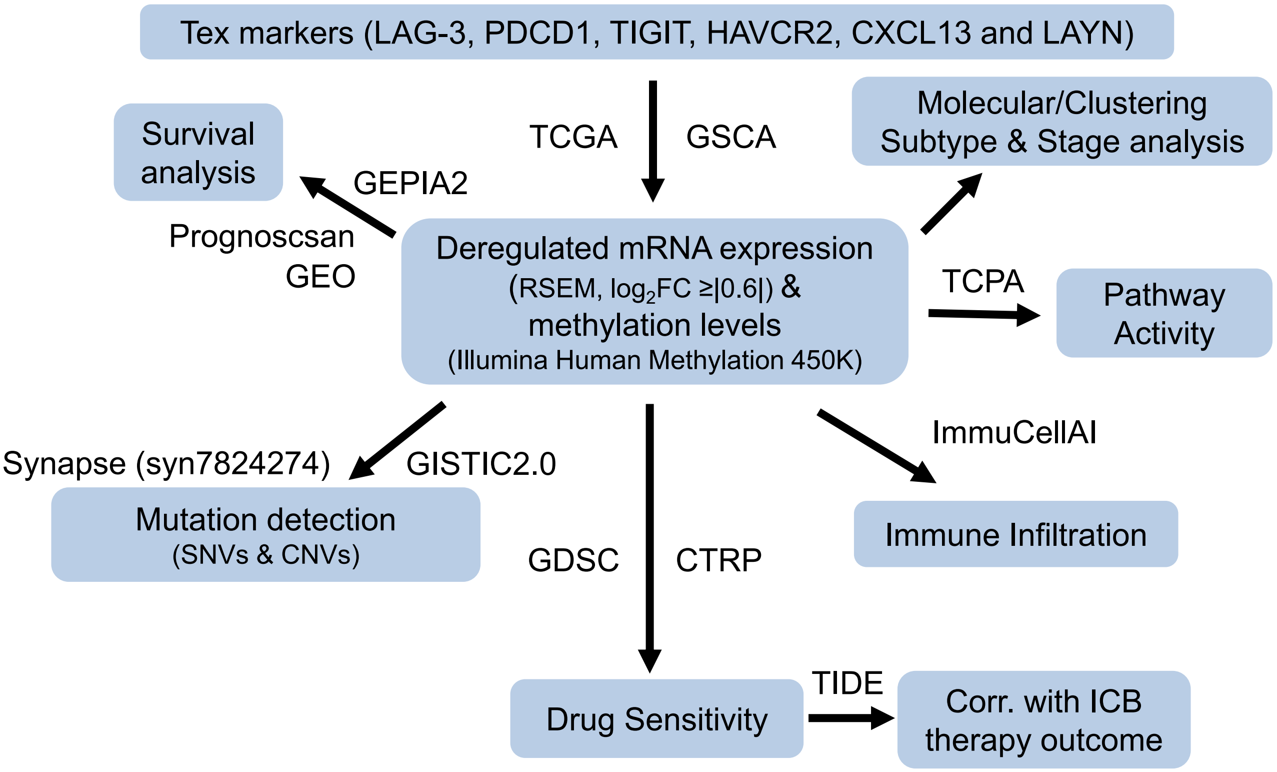
Tex markers (LAG-3, PDCD1, TIGIT, HAVCR2, CXCL13 and LAYN)
TCGA
GSCA
Survival analysis
GEPIA2
Prognoscsan
GEO
Molecular/Clustering Subtype & Stage analysis
Deregulated mRNA expression
(RSEM, $log_2FC \geq |0.6|$) &
methylation levels
(Illumina Human Methylation 450K)
TCPA
Pathway Activity
ImmuCellAI
Immune Infiltration
Synapse (syn7824274)
GISTIC2.0
Mutation detection
(SNVs & CNVs)
GDSC
CTRP
Drug Sensitivity
TIDE
Corr. with ICB therapy outcome

Figure S2

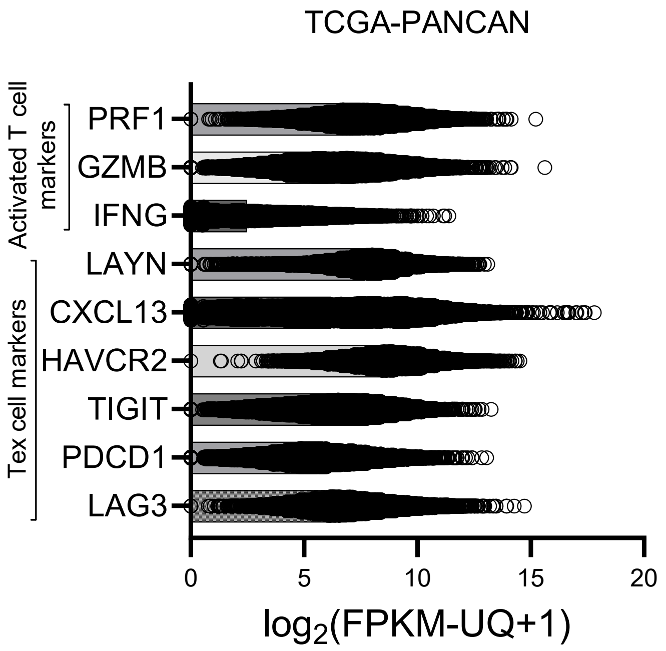

TCGA-PANCAN
Activated T cell markers
PRF1
GZMB
IFNG
Tex cell markers
LAYN
CXCL13
HAVCR2
TIGIT
PDCD1
LAG3
0
5
10
15
20
log2(FPKM-UQ+1)

Figure S3

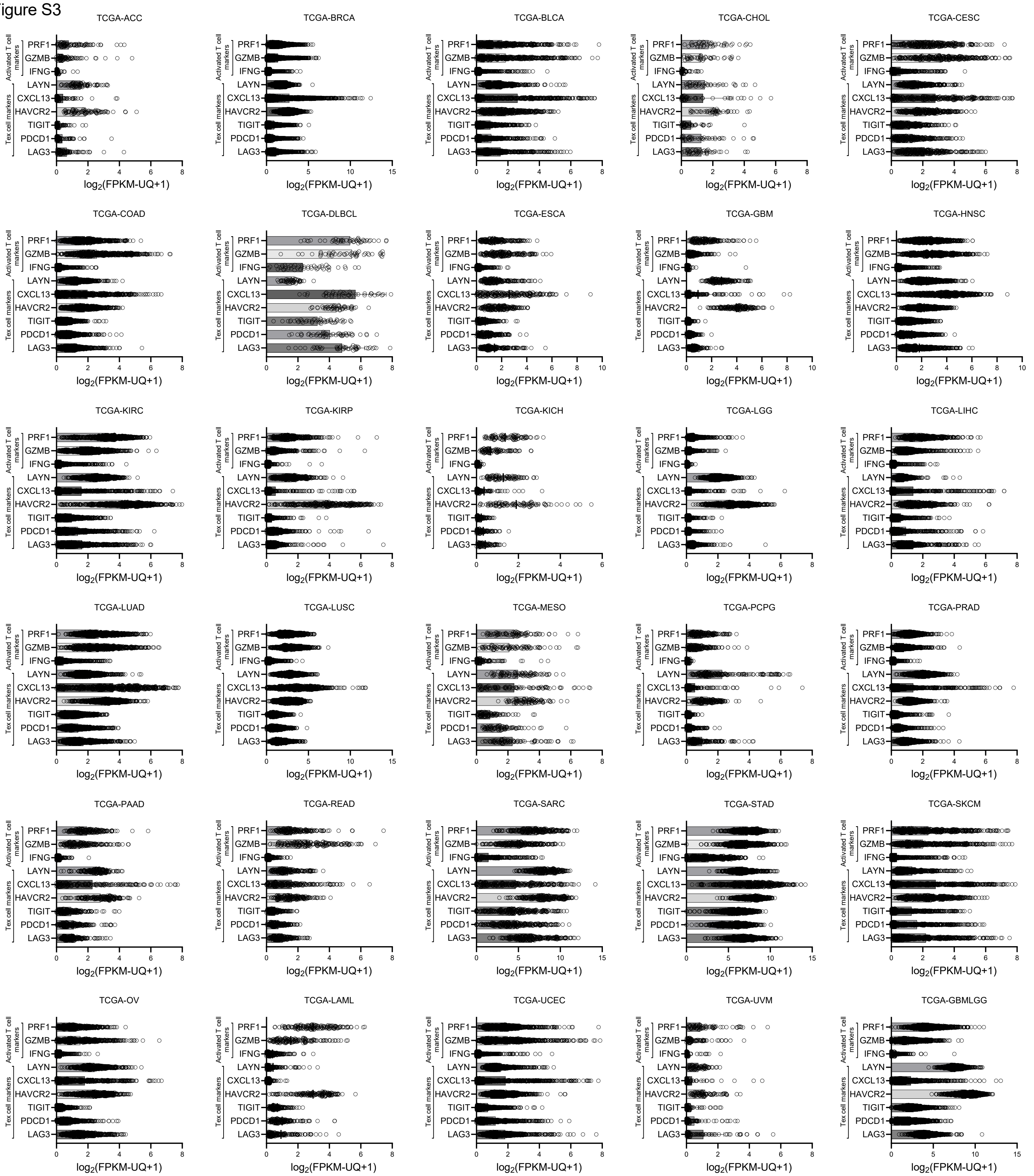

TCGA-ACC
TCGA-BRCA
TCGA-BLCA
TCGA-CHOL
TCGA-CESC
TCGA-COAD
TCGA-DLBCL
TCGA-ESCA
TCGA-GBM
TCGA-HNSC
TCGA-KIRC
TCGA-KIRP
TCGA-KICH
TCGA-LGG
TCGA-LIHC
TCGA-LUAD
TCGA-LUSC
TCGA-MESO
TCGA-PCPG
TCGA-PRAD
TCGA-PAAD
TCGA-READ
TCGA-SARC
TCGA-STAD
TCGA-SKCM
TCGA-OV
TCGA-LAML
TCGA-UCEC
TCGA-UVM
TCGA-GBMLGG
Activated T cell markers
Tex cell markers
PRF1
GZMB
IFNG
LAYN
CXCL13
HAVCR2
TIGIT
PDCD1
LAG3
$\log_2$(FPKM-UQ+1)

Figure S4

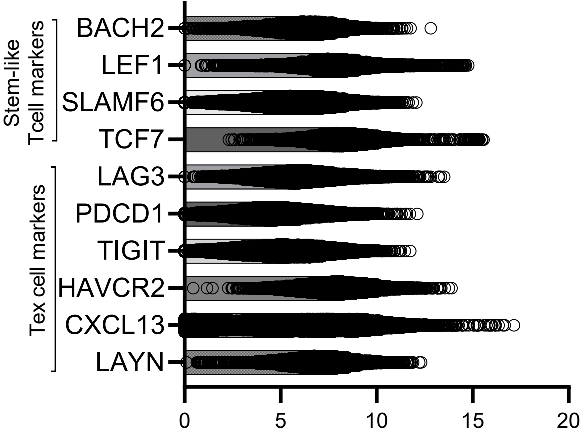
Stem-like Tcell markers
Tex cell markers
BACH2
LEF1
SLAMF6
TCF7
LAG3
PDCD1
TIGIT
HAVCR2
CXCL13
LAYN
0
5
10
15
20

Figure S5(A)

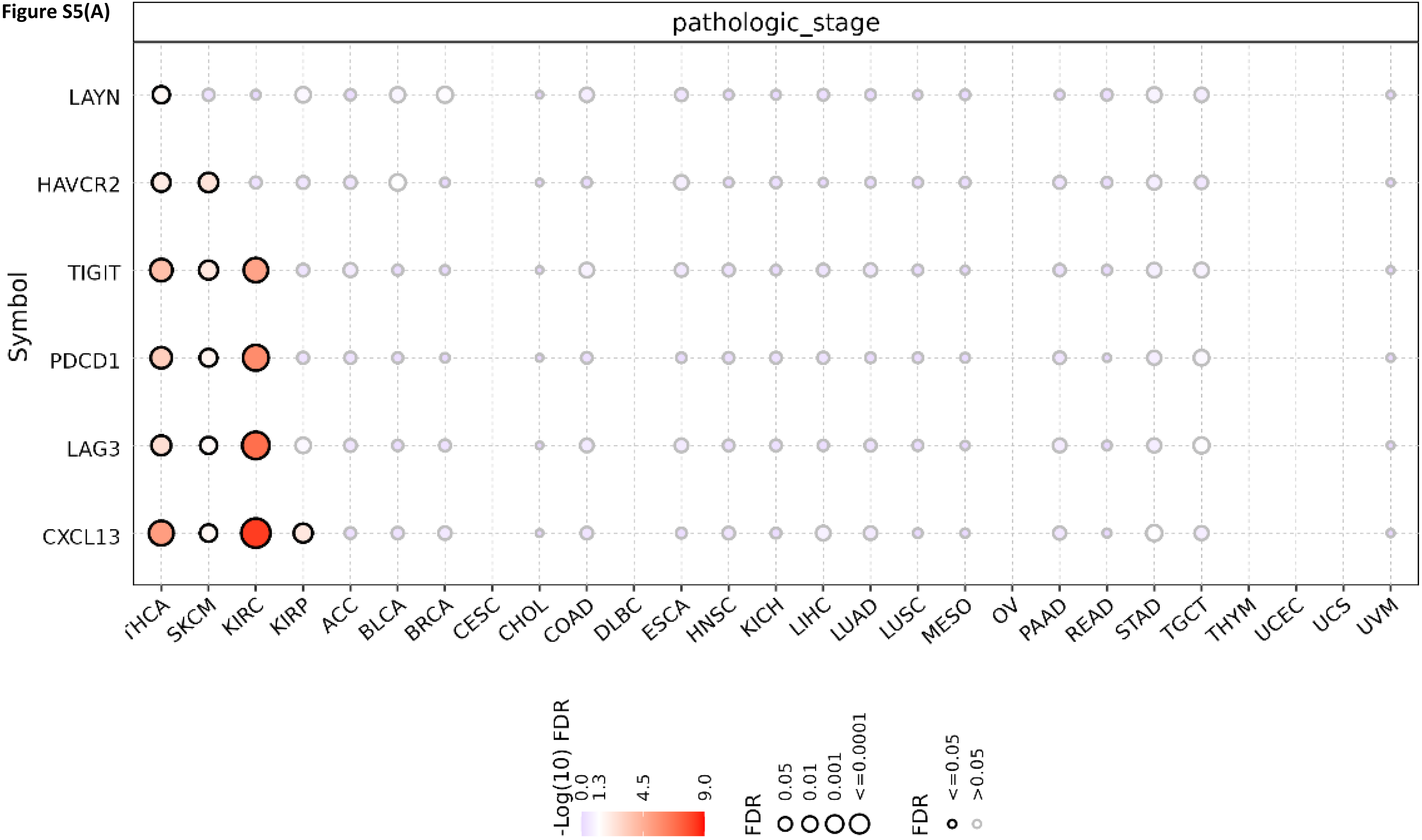
pathologic_stage
Symbol
LAYN
HAVCR2
TIGIT
PDCD1
LAG3
CXCL13
THCA
SKCM
KIRC
KIRP
ACC
BLCA
BRCA
CESC
CHOL
COAD
DLBC
ESCA
HNSC
KICH
LIHC
LUAD
LUSC
MESO
OV
PAAD
READ
STAD
TGCT
THYM
UCEC
UCS
UVM
-Log(10) FDR
0.0
1.3
4.5
9.0
FDR
0.05
0.01
0.001
<=0.0001
FDR
<=0.05
>0.05

Figure S5
(B)

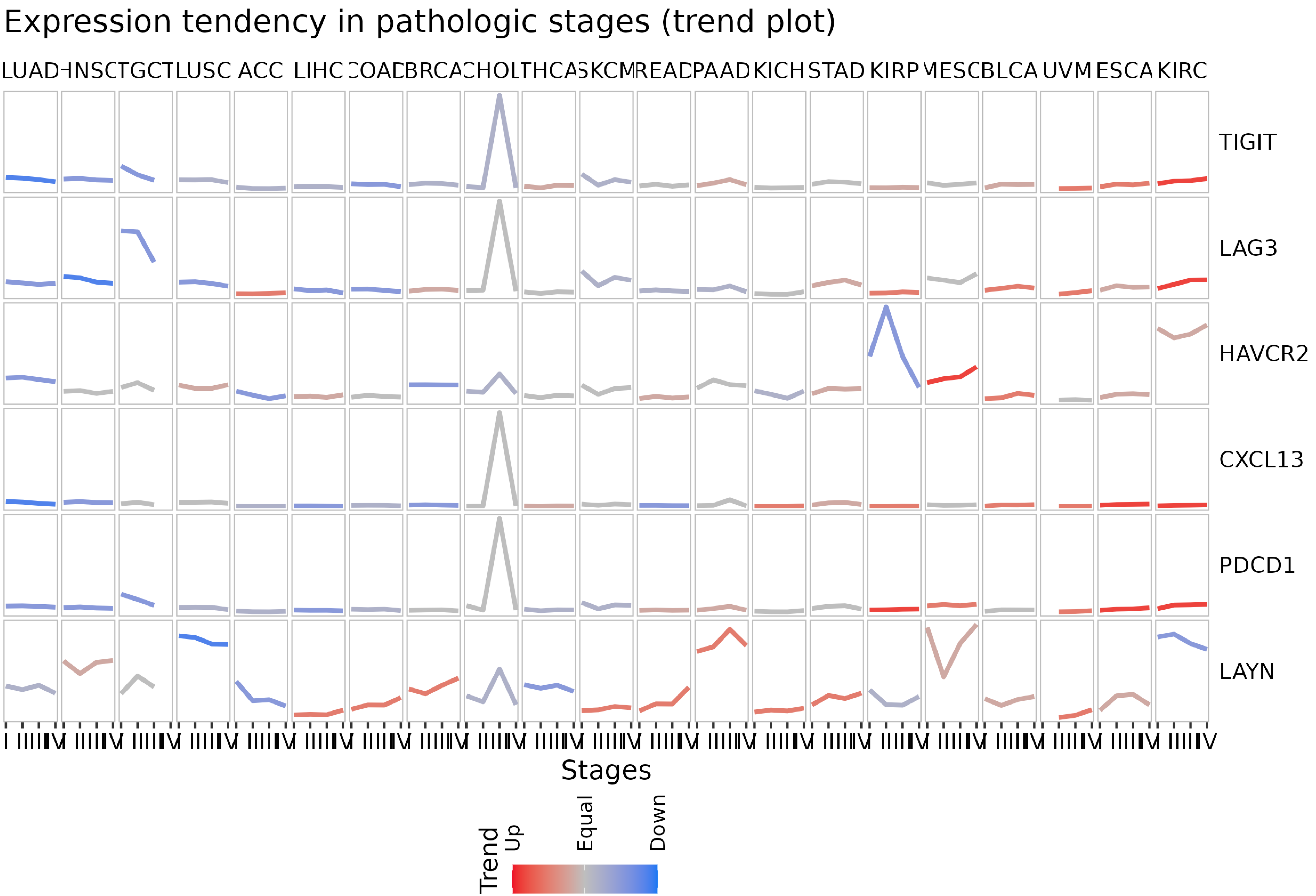

Expression tendency in pathologic stages (trend plot)
LUAD HNSC TGCT LUSC ACC LIHC COAD BRCA CHOL THCA SKCM READ PAAD KICH STAD KIRP MESO BLCA UVM ESCA KIRC
TIGIT
LAG3
HAVCR2
CXCL13
PDCD1
LAYN
Stages
Trend
Up
Equal
Down

Figure S6

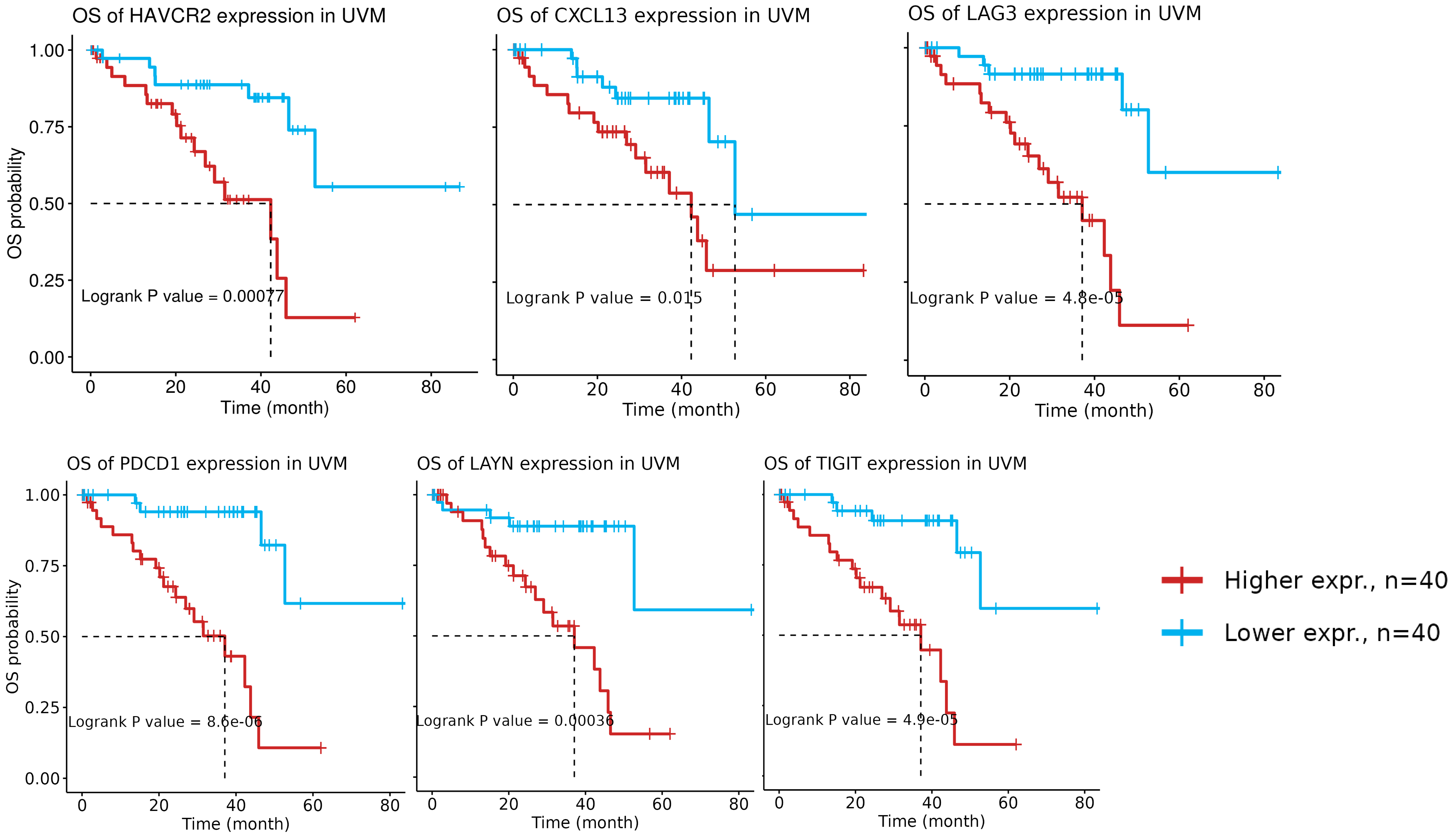

OS of HAVCR2 expression in UVM
OS of CXCL13 expression in UVM
OS of LAG3 expression in UVM
OS of PDCD1 expression in UVM
OS of LAYN expression in UVM
OS of TIGIT expression in UVM
OS probability
1.00
0.75
0.50
0.25
0.00
Time (month)
0
20
40
60
80
Logrank P value = 0.00077
Logrank P value = 0.015
Logrank P value = 4.8e-05
Logrank P value = 8.6e-06
Logrank P value = 0.00036
Logrank P value = 4.9e-05
Higher expr., n=40
Lower expr., n=40

Figure S7

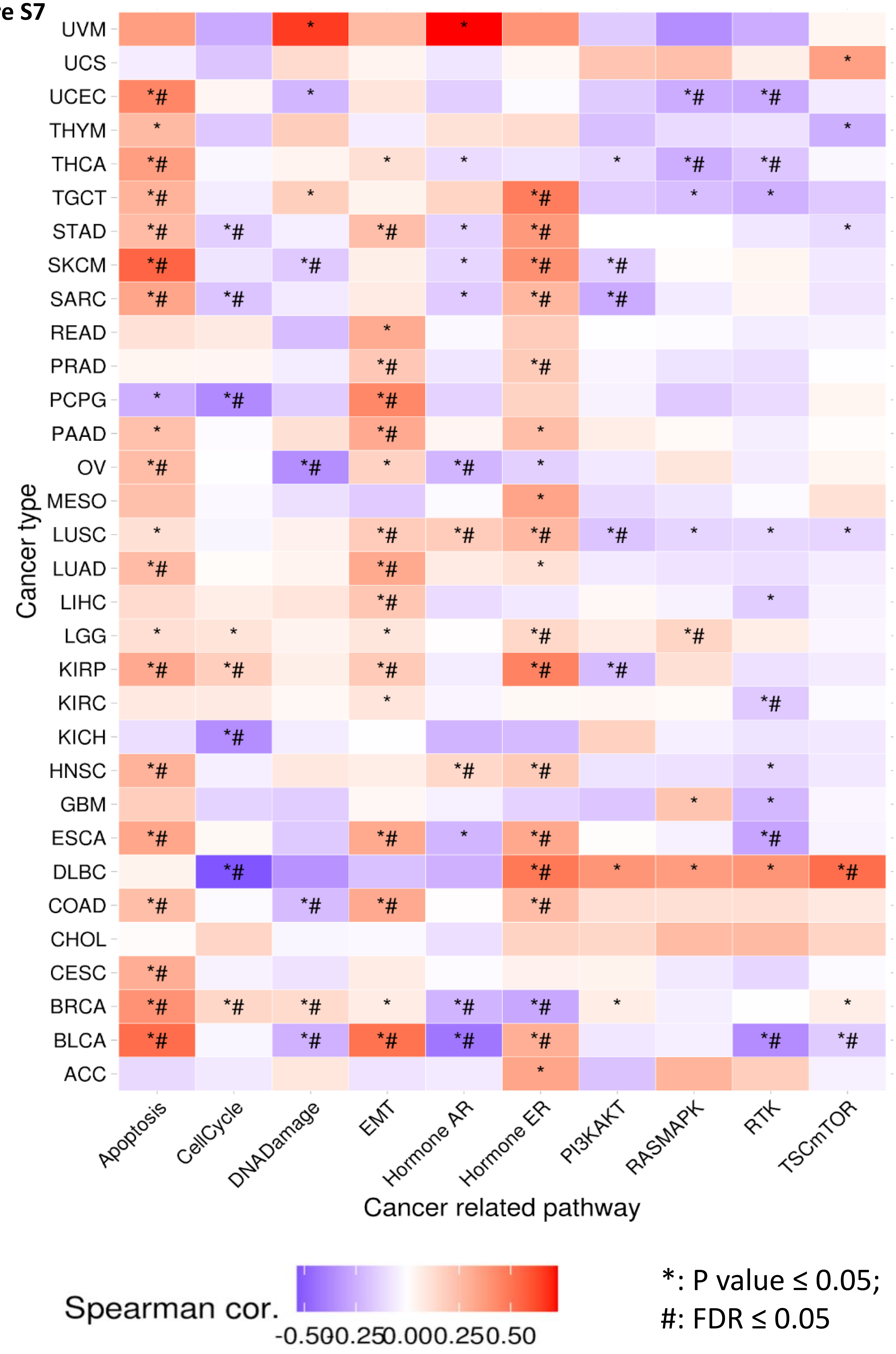
UVM
UCS
UCEC
THYM
THCA
TGCT
STAD
SKCM
SARC
READ
PRAD
PCPG
PAAD
OV
MESO
LUSC
LUAD
LIHC
LGG
KIRP
KIRC
KICH
HNSC
GBM
ESCA
DLBC
COAD
CHOL
CESC
BRCA
BLCA
ACC
Cancer type
Apoptosis
CellCycle
DNADamage
EMT
Hormone AR
Hormone ER
PI3KAKT
RASMAPK
RTK
TSCmTOR
Cancer related pathway
Spearman cor.
-0.50
-0.25
0.00
0.25
0.50
*: P value ≤ 0.05;
#: FDR ≤ 0.05

Figure S8 (A)

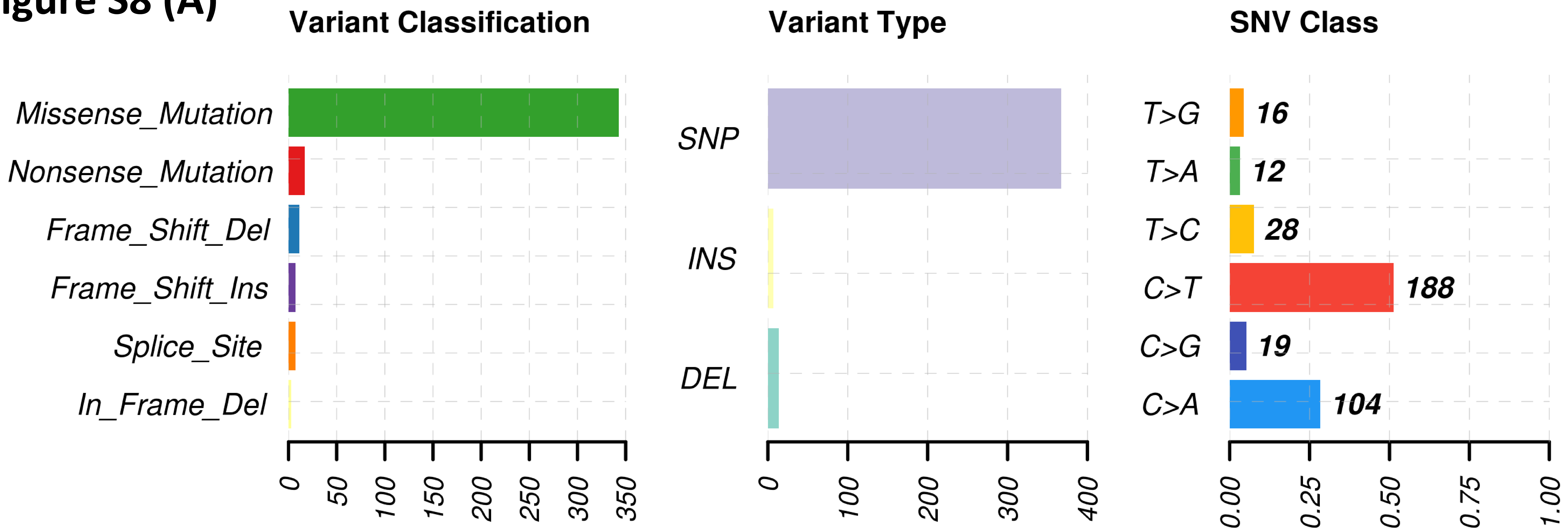

Variant Classification
Missense_Mutation
Nonsense_Mutation
Frame_Shift_Del
Frame_Shift_Ins
Splice_Site
In_Frame_Del
0
50
100
150
200
250
300
350
Variant Type
SNP
INS
DEL
0
100
200
300
400
SNV Class
T>G
T>A
T>C
C>T
C>G
C>A
16
12
28
188
19
104
0.00
0.25
0.50
0.75
1.00

Figure S8 (B)

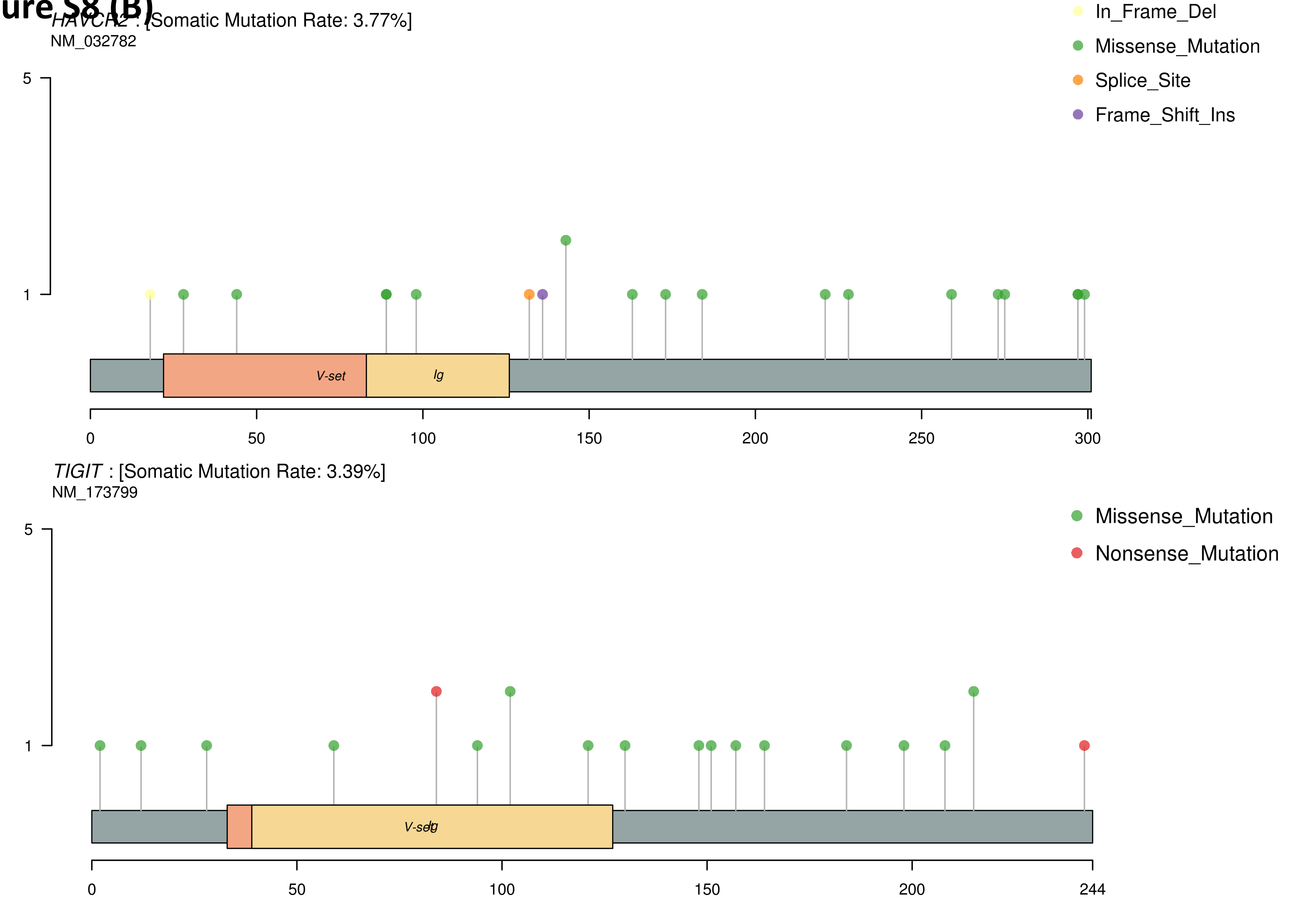

HAVCR2 : [Somatic Mutation Rate: 3.77%]
NM_032782
In_Frame_Del
Missense_Mutation
Splice_Site
Frame_Shift_Ins
5
1
V-set
Ig
0
50
100
150
200
250
300
TIGIT : [Somatic Mutation Rate: 3.39%]
NM_173799
Missense_Mutation
Nonsense_Mutation
5
1
V-set
Ig
0
50
100
150
200
244

Figure S8 (C)

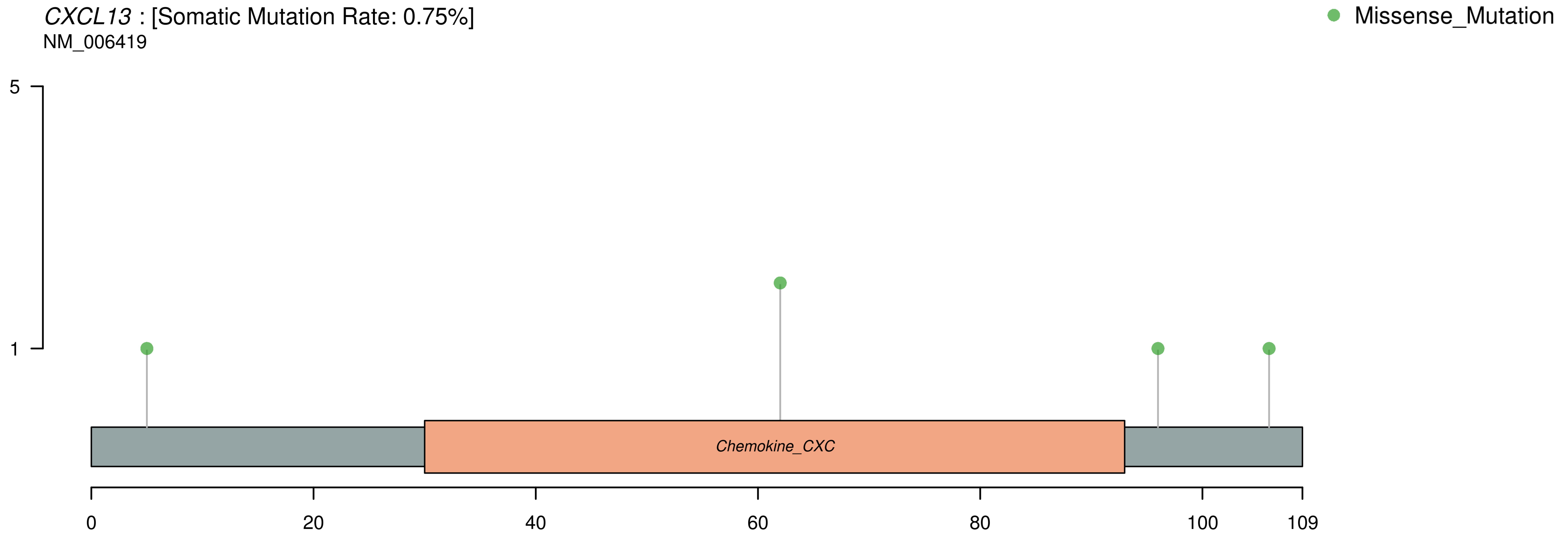
CXCL13 : [Somatic Mutation Rate: 0.75%]
NM_006419
Missense_Mutation
5
1
Chemokine_CXC
0
20
40
60
80
100
109

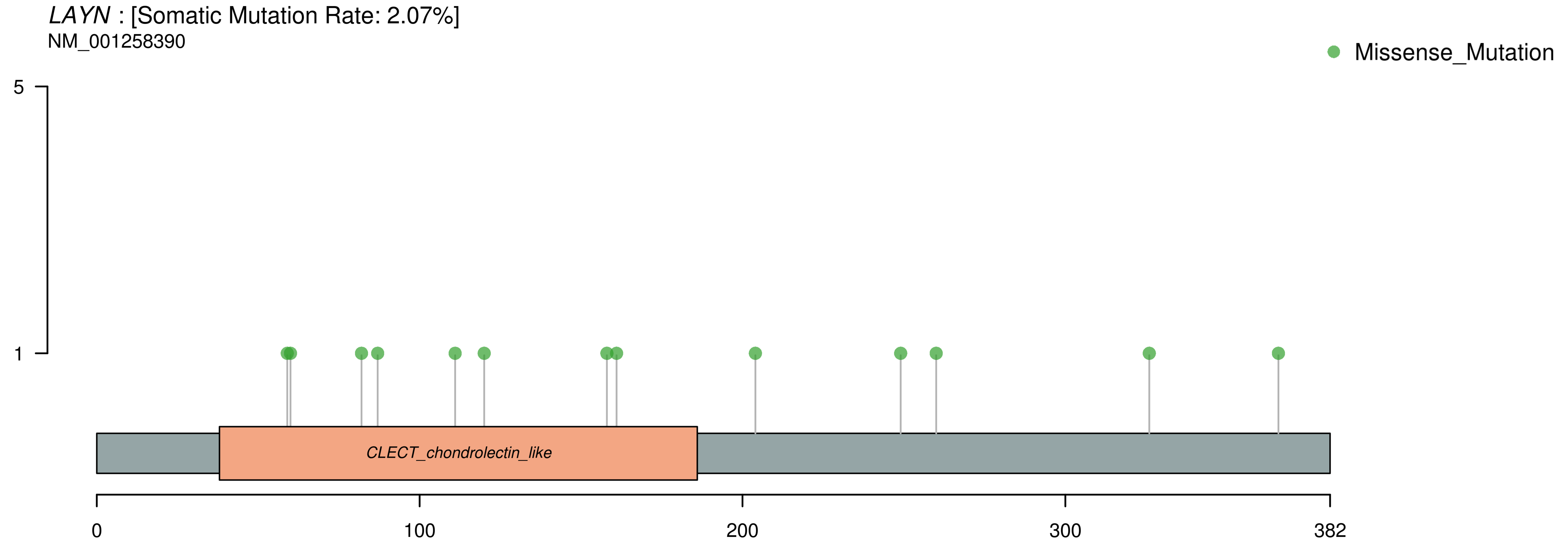
LAYN : [Somatic Mutation Rate: 2.07%]
NM_001258390
Missense_Mutation
5
1
CLECT_chondrolectin_like
0
100
200
300
382

Figure S8 (D)

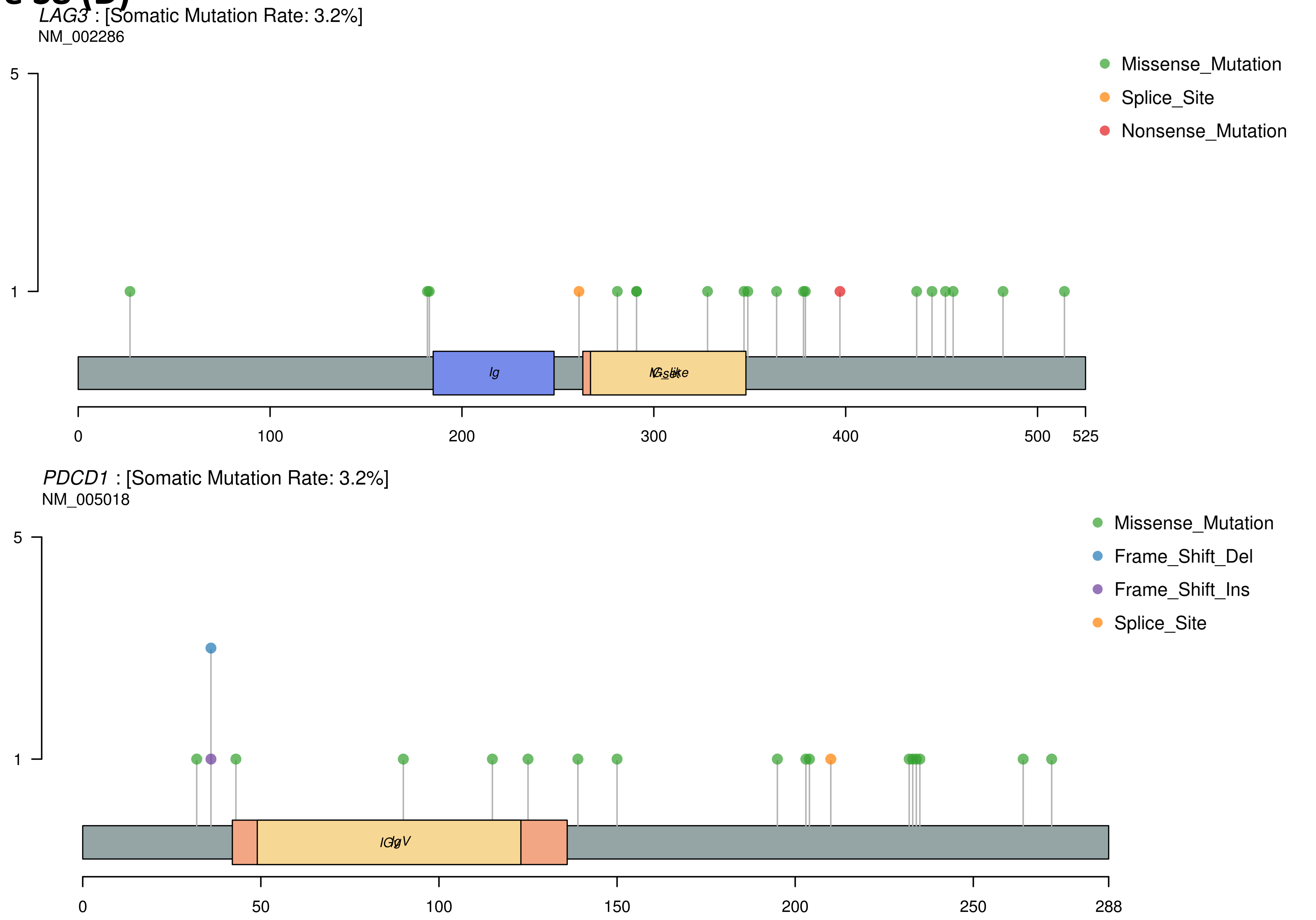
LAG3 : [Somatic Mutation Rate: 3.2%]
NM_002286
5
1
Missense_Mutation
Splice_Site
Nonsense_Mutation
Ig
0
100
200
300
400
500
525
PDCD1 : [Somatic Mutation Rate: 3.2%]
NM_005018
5
1
Missense_Mutation
Frame_Shift_Del
Frame_Shift_Ins
Splice_Site
0
50
100
150
200
250
288

Figure S8 (E)

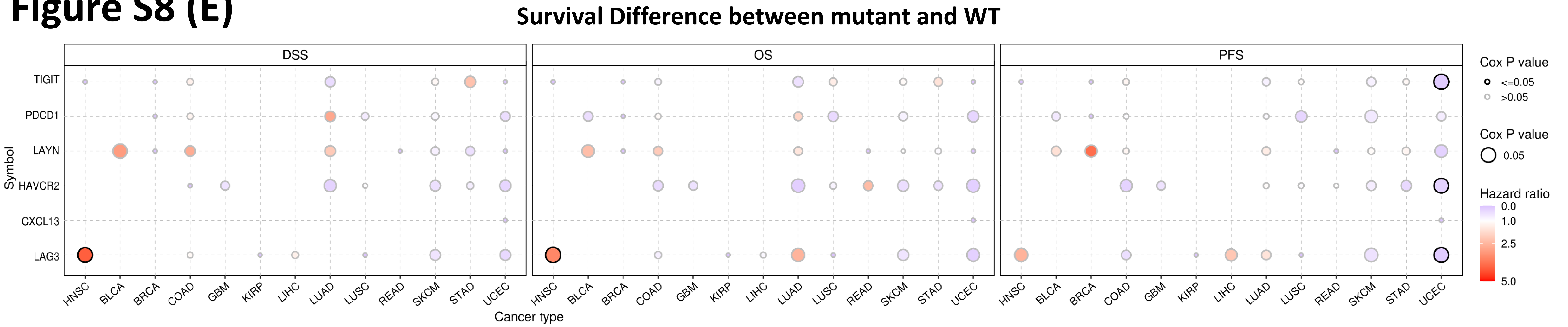

Figure S8 (E)

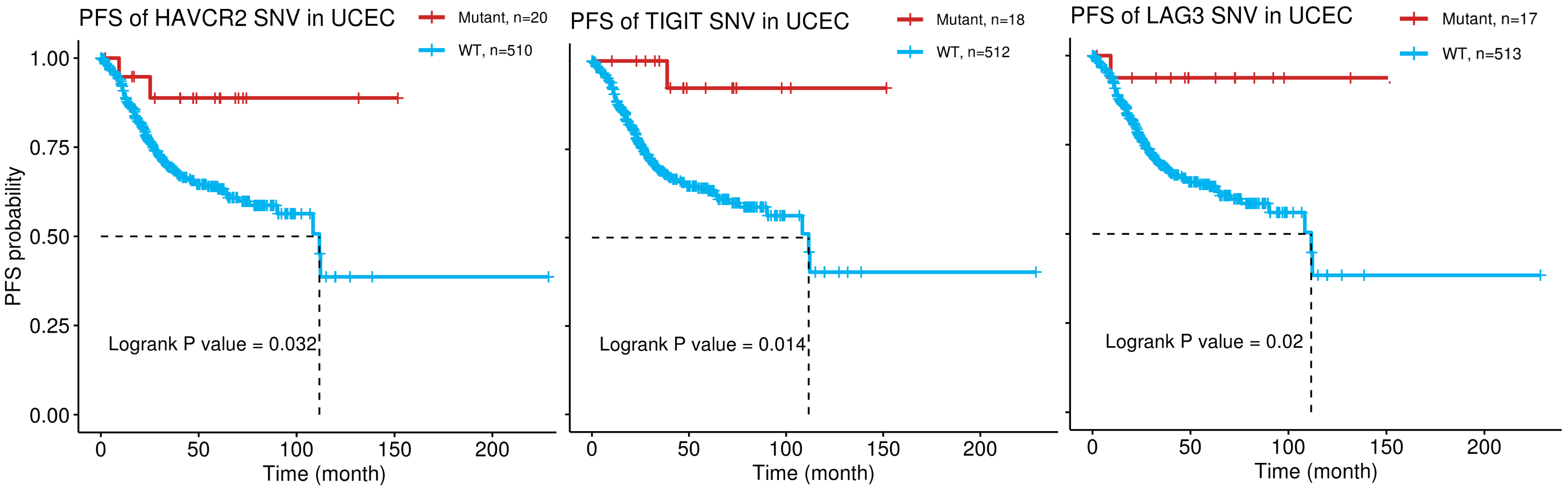

Figure S9(A)

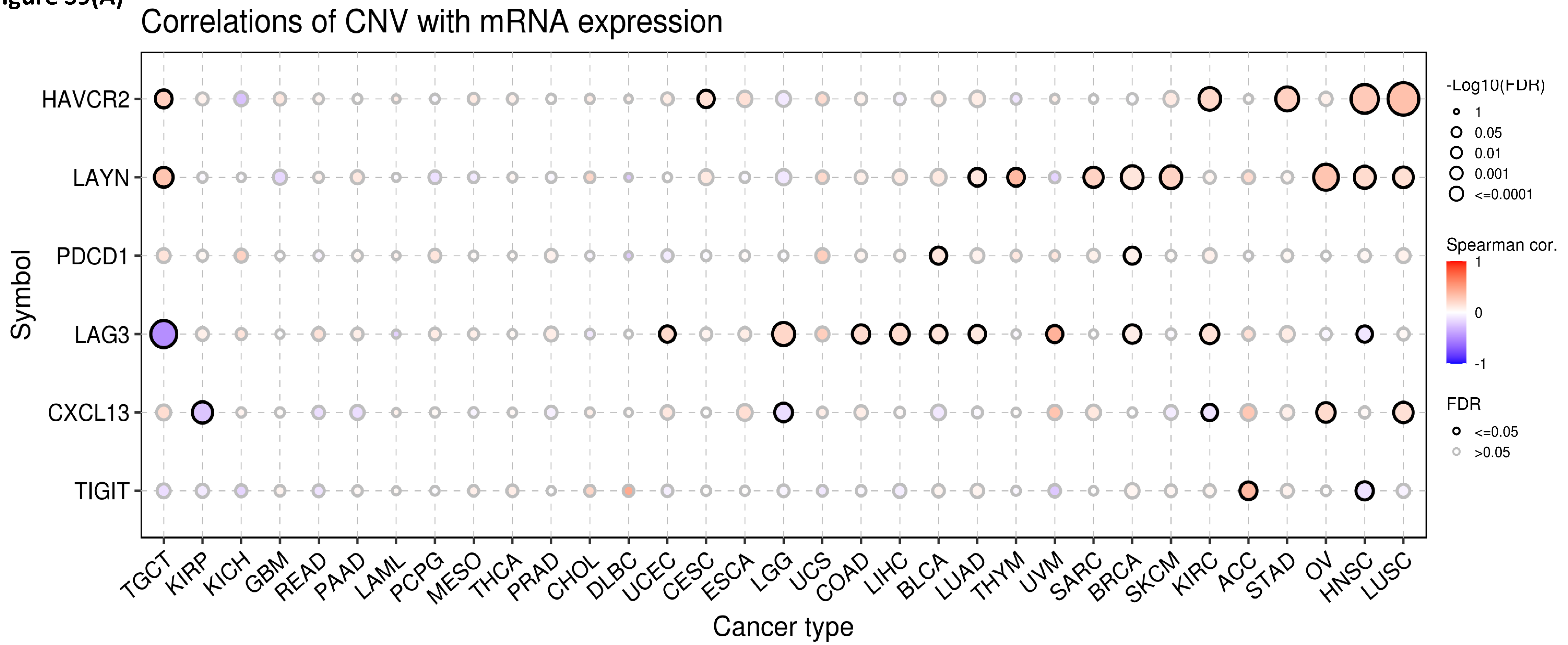
Correlations of CNV with mRNA expression
Symbol
HAVCR2
LAYN
PDCD1
LAG3
CXCL13
TIGIT
Cancer type
TGCT
KIRP
KICH
GBM
READ
PAAD
LAML
PCPG
MESO
THCA
PRAD
CHOL
DLBC
UCEC
CESC
ESCA
LGG
UCS
COAD
LIHC
BLCA
LUAD
THYM
UVM
SARC
BRCA
SKCM
KIRC
ACC
STAD
OV
HNSC
LUSC
-Log10(FDR)
1
0.05
0.01
0.001
<=0.0001
Spearman cor.
1
0
-1
FDR
<=0.05
>0.05

Figure S9(B)

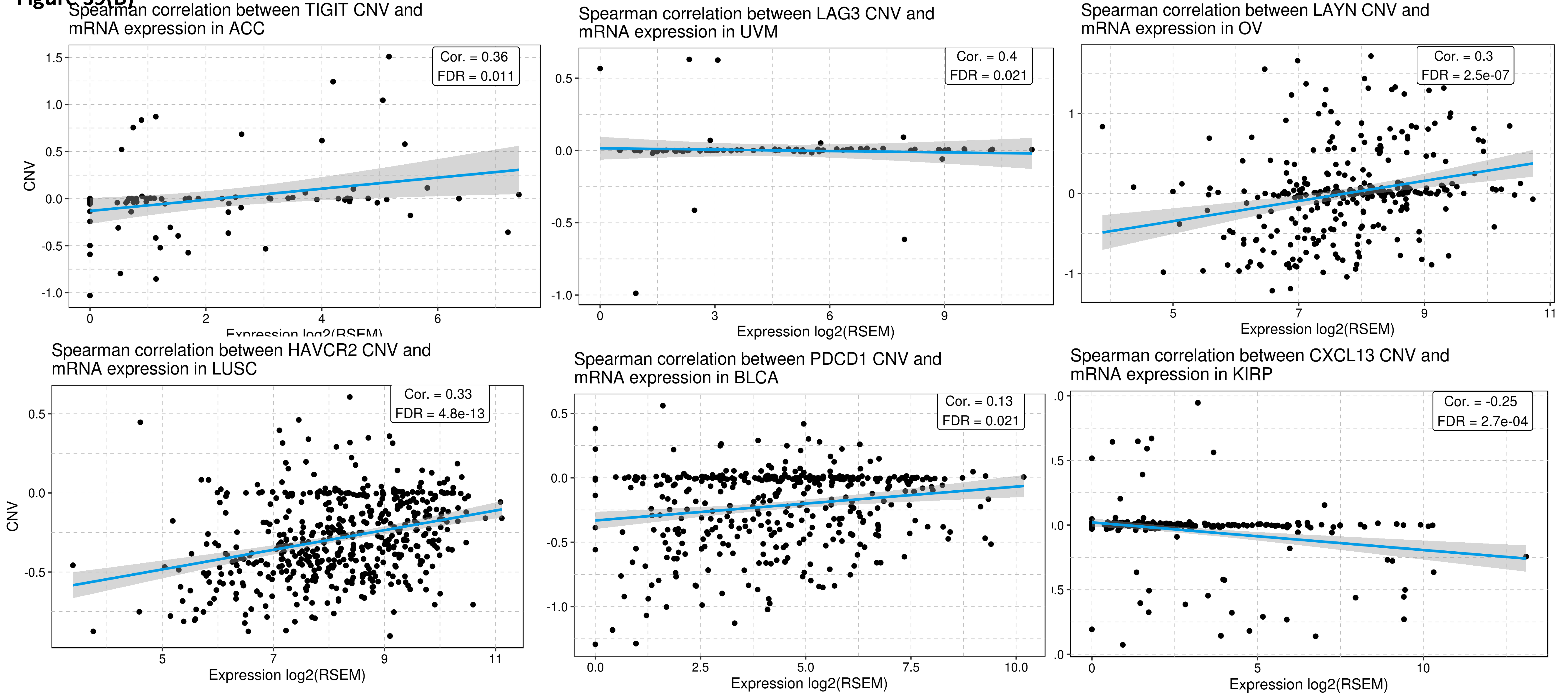
Spearman correlation between TIGIT CNV and mRNA expression in ACC
Cor. = 0.36
FDR = 0.011
CNV
Expression log2(RSEM)
Spearman correlation between LAG3 CNV and mRNA expression in UVM
Cor. = 0.4
FDR = 0.021
Expression log2(RSEM)
Spearman correlation between LAYN CNV and mRNA expression in OV
Cor. = 0.3
FDR = 2.5e-07
Expression log2(RSEM)
Spearman correlation between HAVCR2 CNV and mRNA expression in LUSC
Cor. = 0.33
FDR = 4.8e-13
CNV
Expression log2(RSEM)
Spearman correlation between PDCD1 CNV and mRNA expression in BLCA
Cor. = 0.13
FDR = 0.021
Expression log2(RSEM)
Spearman correlation between CXCL13 CNV and mRNA expression in KIRP
Cor. = -0.25
FDR = 2.7e-04
Expression log2(RSEM)

Figure S9(C)

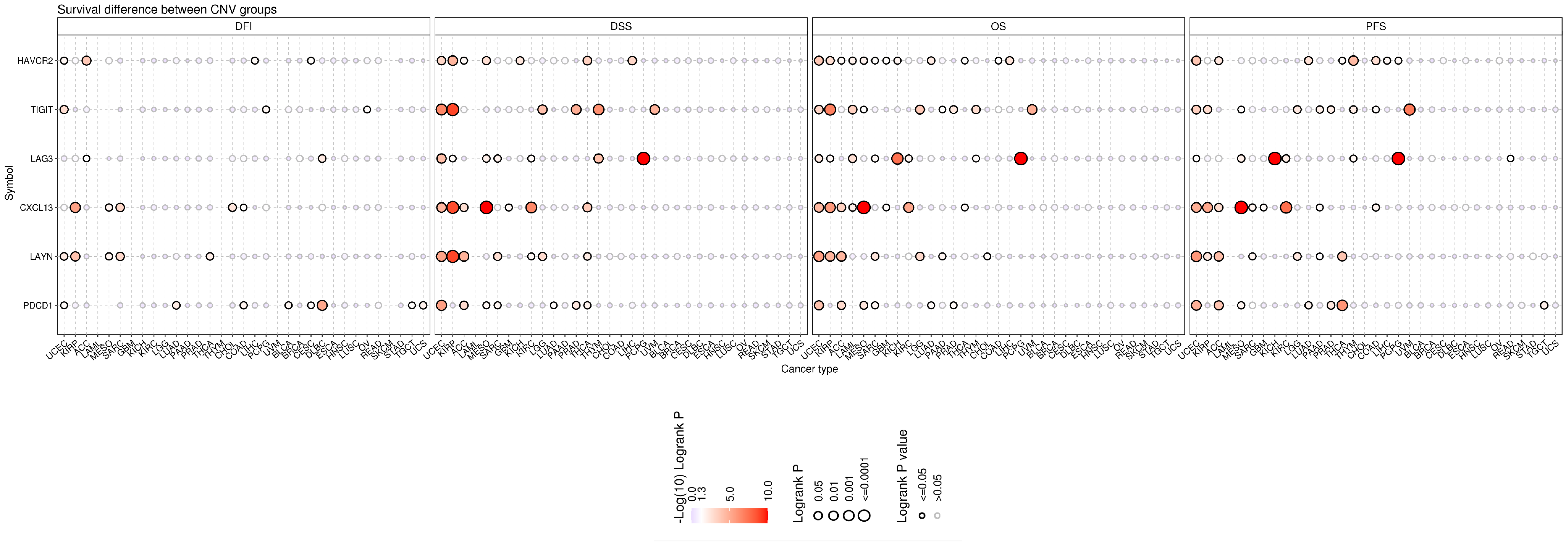
Survival difference between CNV groups
DFI
DSS
OS
PFS
Symbol
HAVCR2
TIGIT
LAG3
CXCL13
LAYN
PDCD1
UCEC KIRP ACC LAML MESO SARC GBM KICH KIRC LGG LUAD PAAD PRAD THCA THYM CHOL COAD LIHC PCPG UVM BLCA BRCA CESC DLBC ESCA HNSC LUSC OV READ SKCM STAD TGCT UCS
Cancer type
-Log(10) Logrank P
0.0
1.3
5.0
10.0
Logrank P
0.05
0.01
0.001
<=0.0001
Logrank P value
<=0.05
>0.05

Figure S9(D)

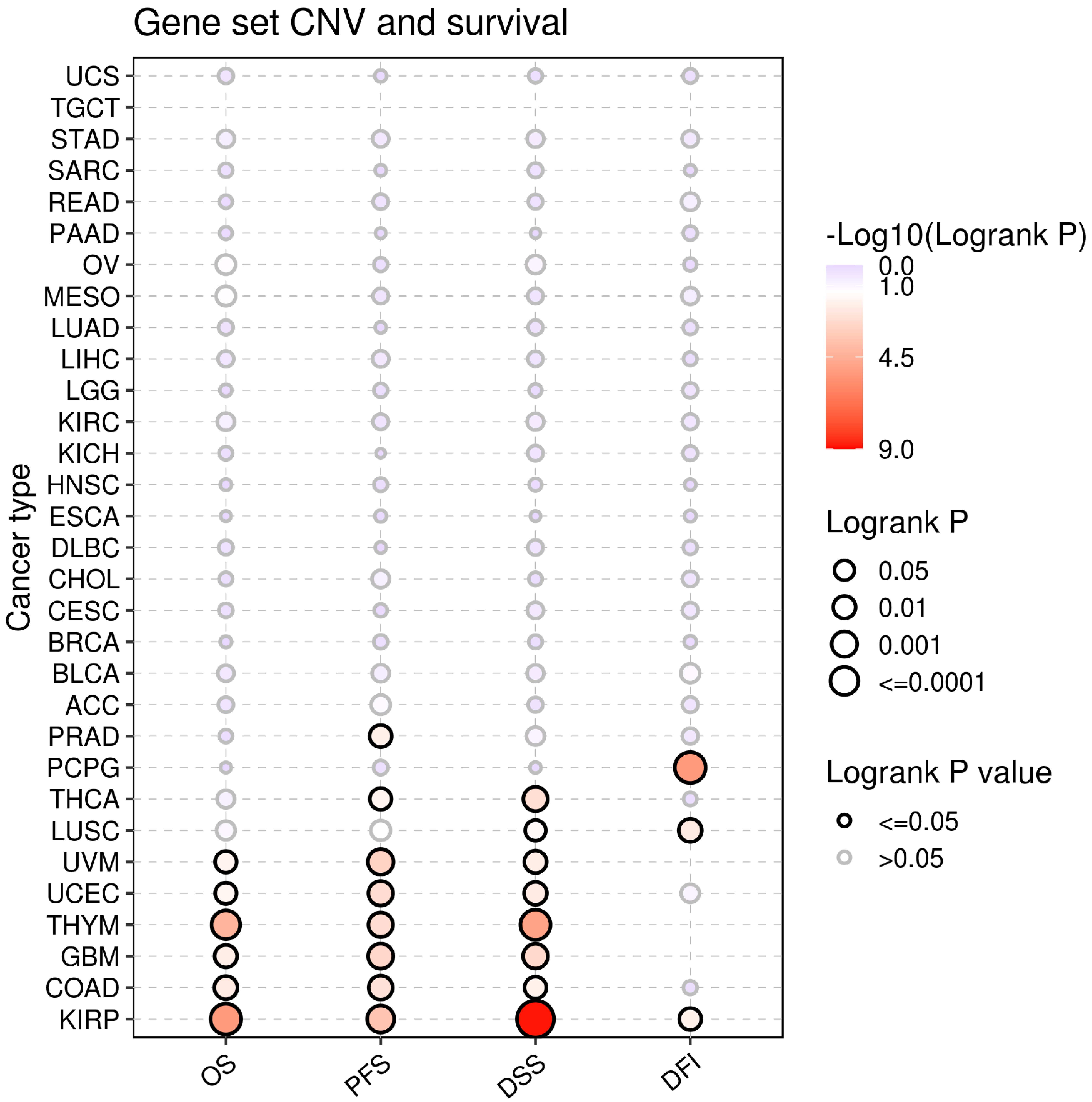

Gene set CNV and survival
UCS
TGCT
STAD
SARC
READ
PAAD
OV
MESO
LUAD
LIHC
LGG
KIRC
KICH
HNSC
ESCA
DLBC
CHOL
CESC
BRCA
BLCA
ACC
PRAD
PCPG
THCA
LUSC
UVM
UCEC
THYM
GBM
COAD
KIRP
Cancer type
OS
PFS
DSS
DFI
-Log10(Logrank P)
0.0
1.0
4.5
9.0
Logrank P
0.05
0.01
0.001
<=0.0001
Logrank P value
<=0.05
>0.05

Figure S9(E)

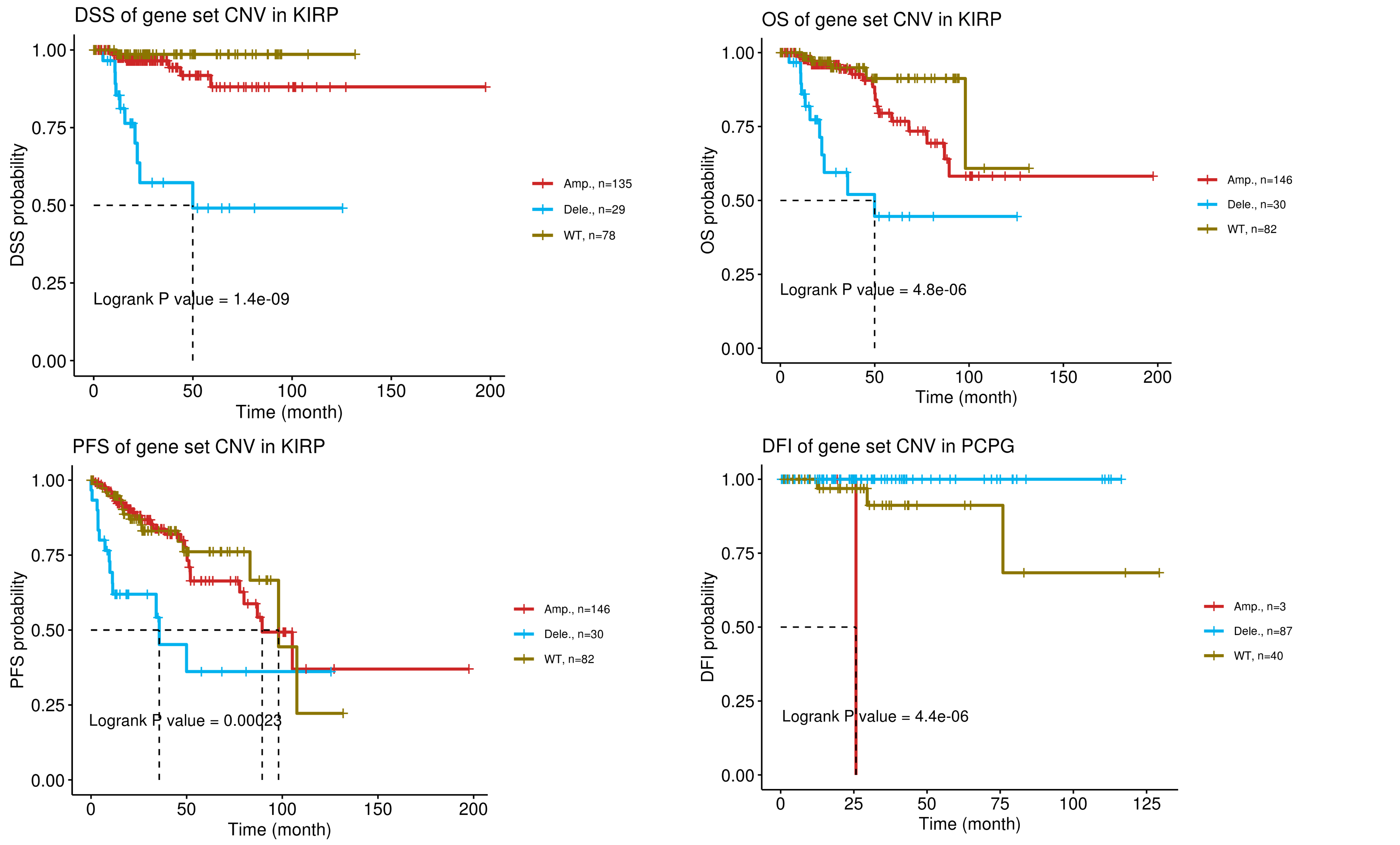
DSS of gene set CNV in KIRP
DSS probability
1.00
0.75
0.50
0.25
0.00
Logrank P value = 1.4e-09
0
50
100
150
200
Time (month)
Amp., n=135
Dele., n=29
WT, n=78
OS of gene set CNV in KIRP
OS probability
Logrank P value = 4.8e-06
Amp., n=146
Dele., n=30
WT, n=82
PFS of gene set CNV in KIRP
PFS probability
Logrank P value = 0.00023
Amp., n=146
Dele., n=30
WT, n=82
DFI of gene set CNV in PCPG
DFI probability
Logrank P value = 4.4e-06
25
75
125
Amp., n=3
Dele., n=87
WT, n=40

Figure S10(A)

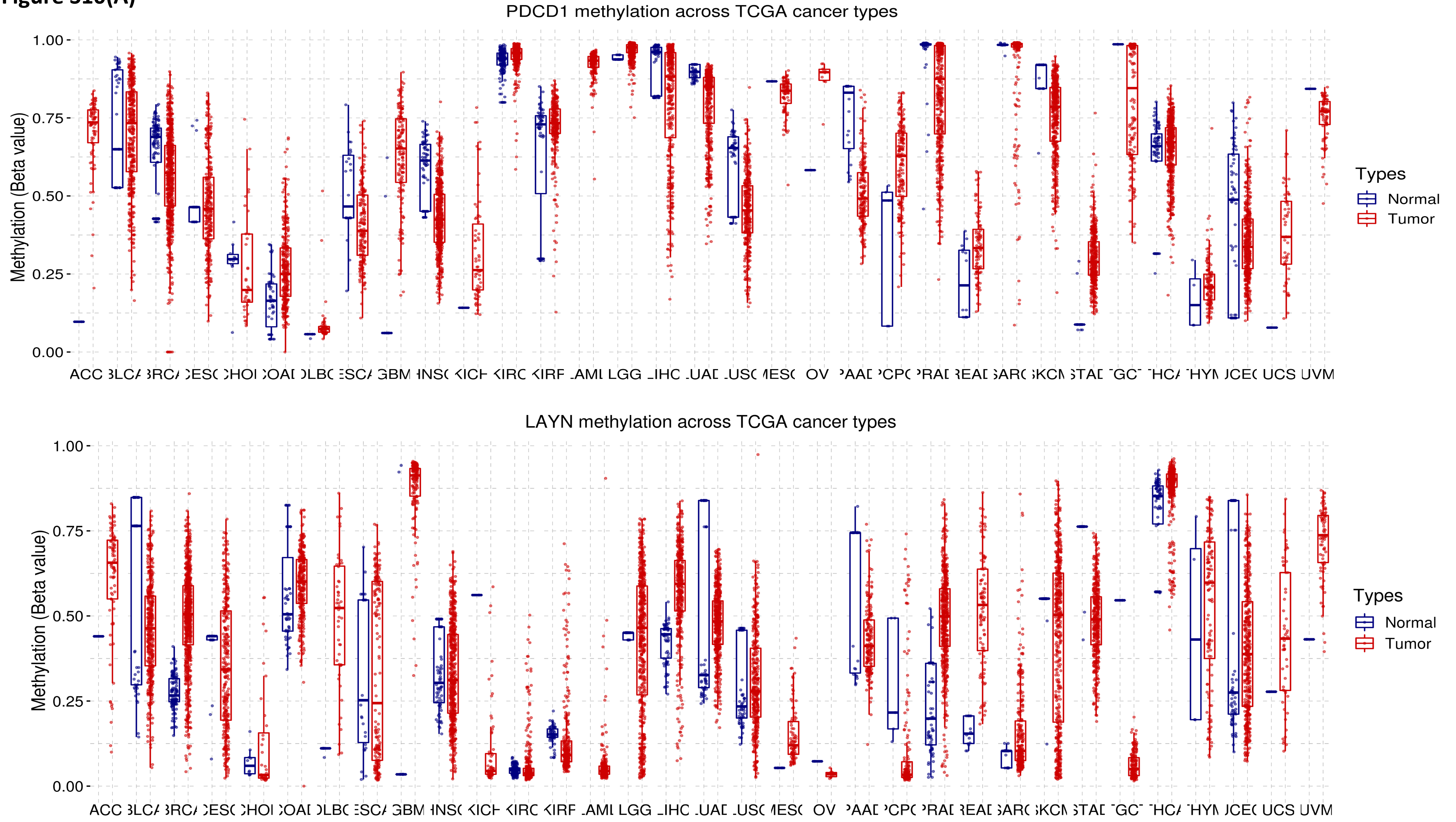

PDCD1 methylation across TCGA cancer types
Methylation (Beta value)
1.00
0.75
0.50
0.25
0.00
ACC
UCS
UVM
OV
LGG
GBM
Types
Normal
Tumor
LAYN methylation across TCGA cancer types

Figure S10(B)

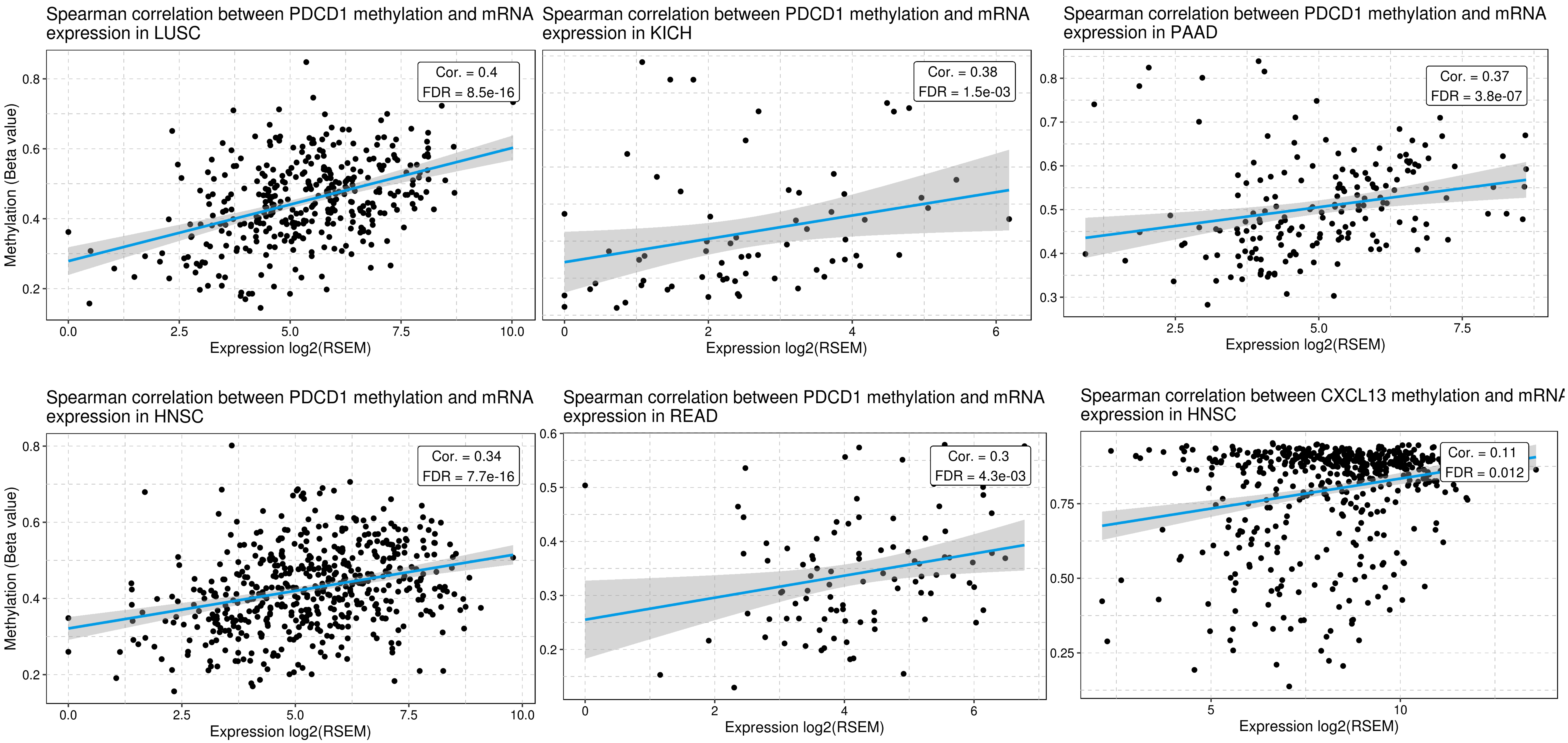
Spearman correlation between PDCD1 methylation and mRNA expression in LUSC
Cor. = 0.4
FDR = 8.5e-16
Methylation (Beta value)
0.2
0.4
0.6
0.8
0.0
2.5
5.0
7.5
10.0
Expression log2(RSEM)
Spearman correlation between PDCD1 methylation and mRNA expression in KICH
Cor. = 0.38
FDR = 1.5e-03
0
2
4
6
Expression log2(RSEM)
Spearman correlation between PDCD1 methylation and mRNA expression in PAAD
Cor. = 0.37
FDR = 3.8e-07
0.3
0.4
0.5
0.6
0.7
0.8
2.5
5.0
7.5
Expression log2(RSEM)
Spearman correlation between PDCD1 methylation and mRNA expression in HNSC
Cor. = 0.34
FDR = 7.7e-16
Methylation (Beta value)
0.2
0.4
0.6
0.8
0.0
2.5
5.0
7.5
10.0
Expression log2(RSEM)
Spearman correlation between PDCD1 methylation and mRNA expression in READ
Cor. = 0.3
FDR = 4.3e-03
0.2
0.3
0.4
0.5
0.6
0
2
4
6
Expression log2(RSEM)
Spearman correlation between CXCL13 methylation and mRNA expression in HNSC
Cor. = 0.11
FDR = 0.012
0.25
0.50
0.75
5
10
Expression log2(RSEM)

Figure S10(C)

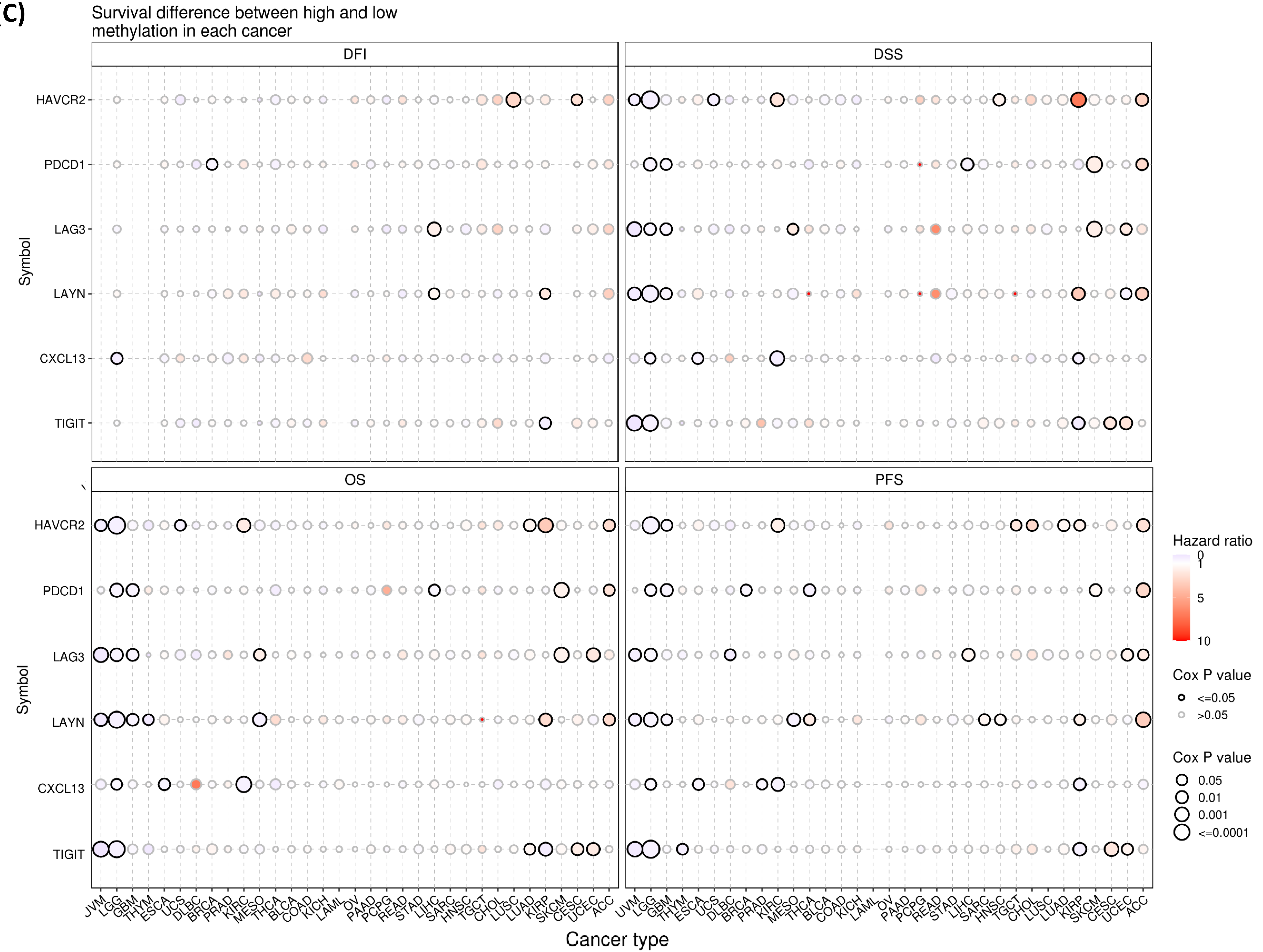
Survival difference between high and low methylation in each cancer
DFI
DSS
OS
PFS
Symbol
HAVCR2
PDCD1
LAG3
LAYN
CXCL13
TIGIT
UVM
LGG
GBM
THYM
ESCA
UCS
DLBC
BRCA
PRAD
KIRC
MESO
THCA
BLCA
COAD
KICH
LAML
OV
PAAD
PCPG
READ
STAD
LIHC
SARC
HNSC
TGCT
CHOL
LUSC
LUAD
KIRP
SKCM
CESC
UCEC
ACC
Cancer type
Hazard ratio
0
1
5
10
Cox P value
<=0.05
>0.05
Cox P value
0.05
0.01
0.001
<=0.0001

Figure S10(D)

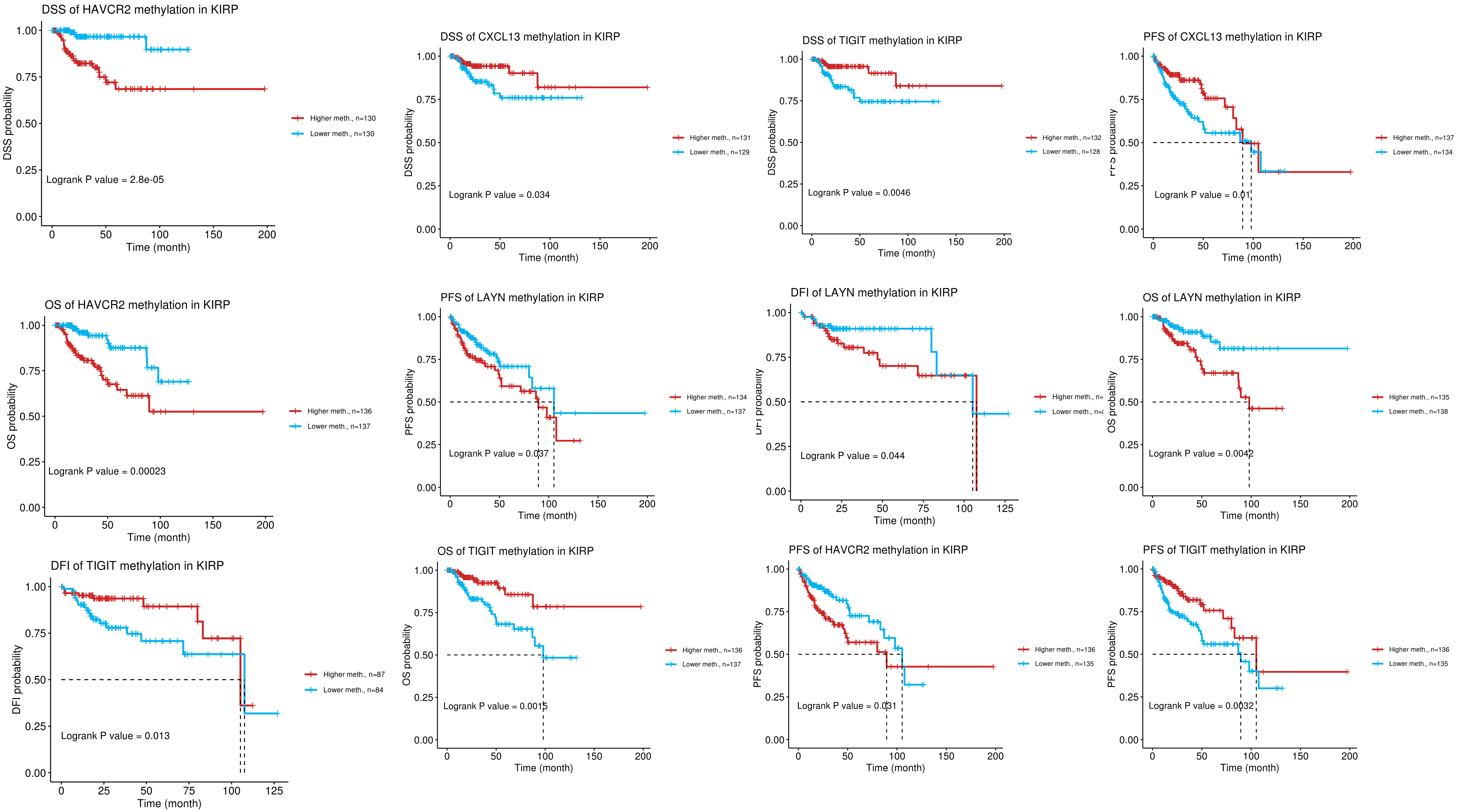
DSS of HAVCR2 methylation in KIRP
Higher meth., n=130
Lower meth., n=130
Logrank P value = 2.8e-05
DSS of CXCL13 methylation in KIRP
Higher meth., n=131
Lower meth., n=129
Logrank P value = 0.034
DSS of TIGIT methylation in KIRP
Higher meth., n=132
Lower meth., n=128
Logrank P value = 0.0046
PFS of CXCL13 methylation in KIRP
Higher meth., n=137
Lower meth., n=134
Logrank P value = 0.01
OS of HAVCR2 methylation in KIRP
Higher meth., n=136
Lower meth., n=137
Logrank P value = 0.00023
PFS of LAYN methylation in KIRP
Higher meth., n=134
Lower meth., n=137
Logrank P value = 0.037
DFI of LAYN methylation in KIRP
Logrank P value = 0.044
OS of LAYN methylation in KIRP
Higher meth., n=135
Lower meth., n=138
Logrank P value = 0.0042
DFI of TIGIT methylation in KIRP
Higher meth., n=87
Lower meth., n=84
Logrank P value = 0.013
OS of TIGIT methylation in KIRP
Higher meth., n=136
Lower meth., n=137
Logrank P value = 0.0015
PFS of HAVCR2 methylation in KIRP
Higher meth., n=136
Lower meth., n=135
Logrank P value = 0.031
PFS of TIGIT methylation in KIRP
Higher meth., n=136
Lower meth., n=135
Logrank P value = 0.0032
DSS probability
OS probability
PFS probability
DFI probability
Time (month)

Figure S11 (A)

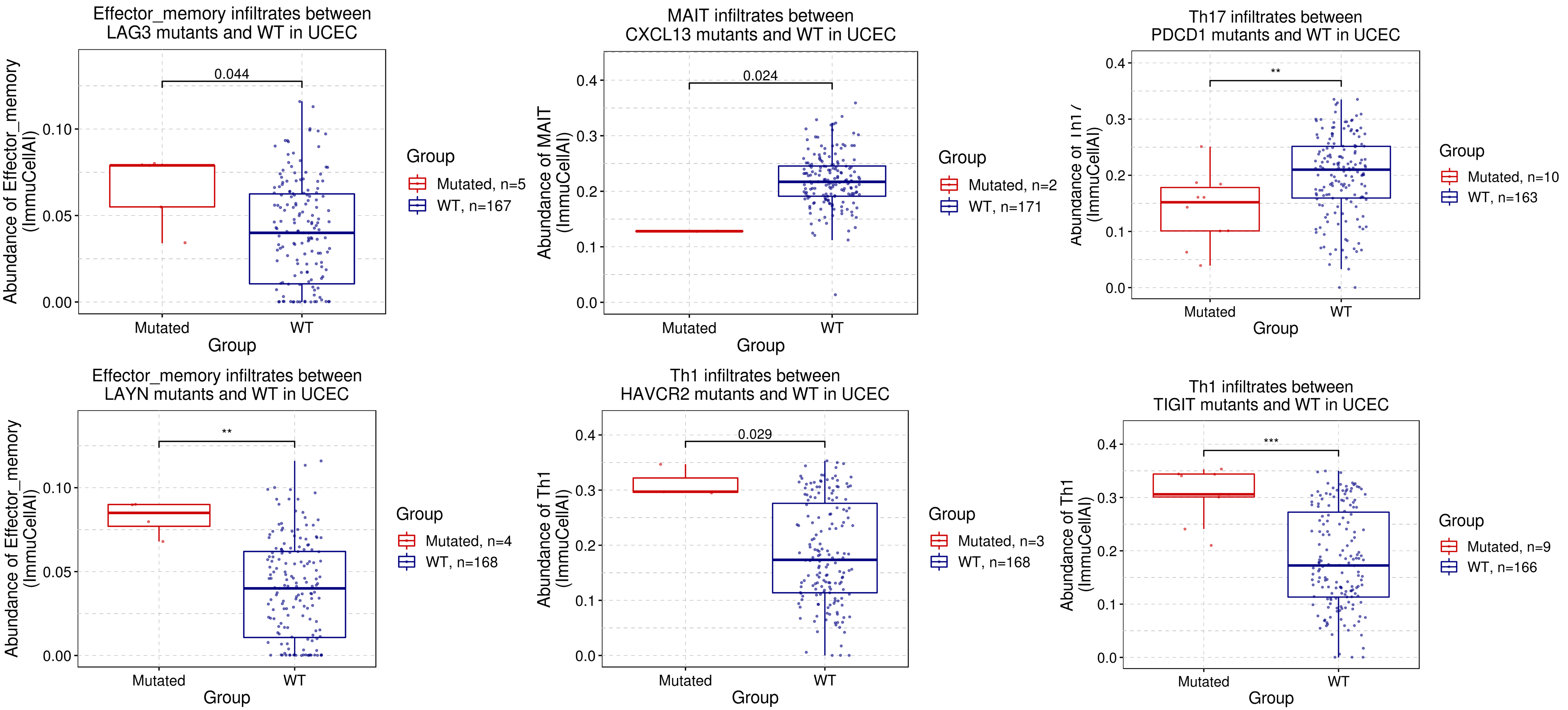
Effector_memory infiltrates between LAG3 mutants and WT in UCEC
0.044
Abundance of Effector_memory (ImmuCellAI)
0.10
0.05
0.00
Mutated
WT
Group
Group
Mutated, n=5
WT, n=167
MAIT infiltrates between CXCL13 mutants and WT in UCEC
0.024
Abundance of MAIT (ImmuCellAI)
0.4
0.3
0.2
0.1
0.0
Mutated
WT
Group
Group
Mutated, n=2
WT, n=171
Th17 infiltrates between PDCD1 mutants and WT in UCEC
**
Abundance of Th17 (ImmuCellAI)
0.4
0.3
0.2
0.1
0.0
Mutated
WT
Group
Group
Mutated, n=10
WT, n=163
Effector_memory infiltrates between LAYN mutants and WT in UCEC
**
Abundance of Effector_memory (ImmuCellAI)
0.10
0.05
0.00
Mutated
WT
Group
Group
Mutated, n=4
WT, n=168
Th1 infiltrates between HAVCR2 mutants and WT in UCEC
0.029
Abundance of Th1 (ImmuCellAI)
0.4
0.3
0.2
0.1
0.0
Mutated
WT
Group
Group
Mutated, n=3
WT, n=168
Th1 infiltrates between TIGIT mutants and WT in UCEC
***
Abundance of Th1 (ImmuCellAI)
0.4
0.3
0.2
0.1
0.0
Mutated
WT
Group
Group
Mutated, n=9
WT, n=166

Figure S11 (B)

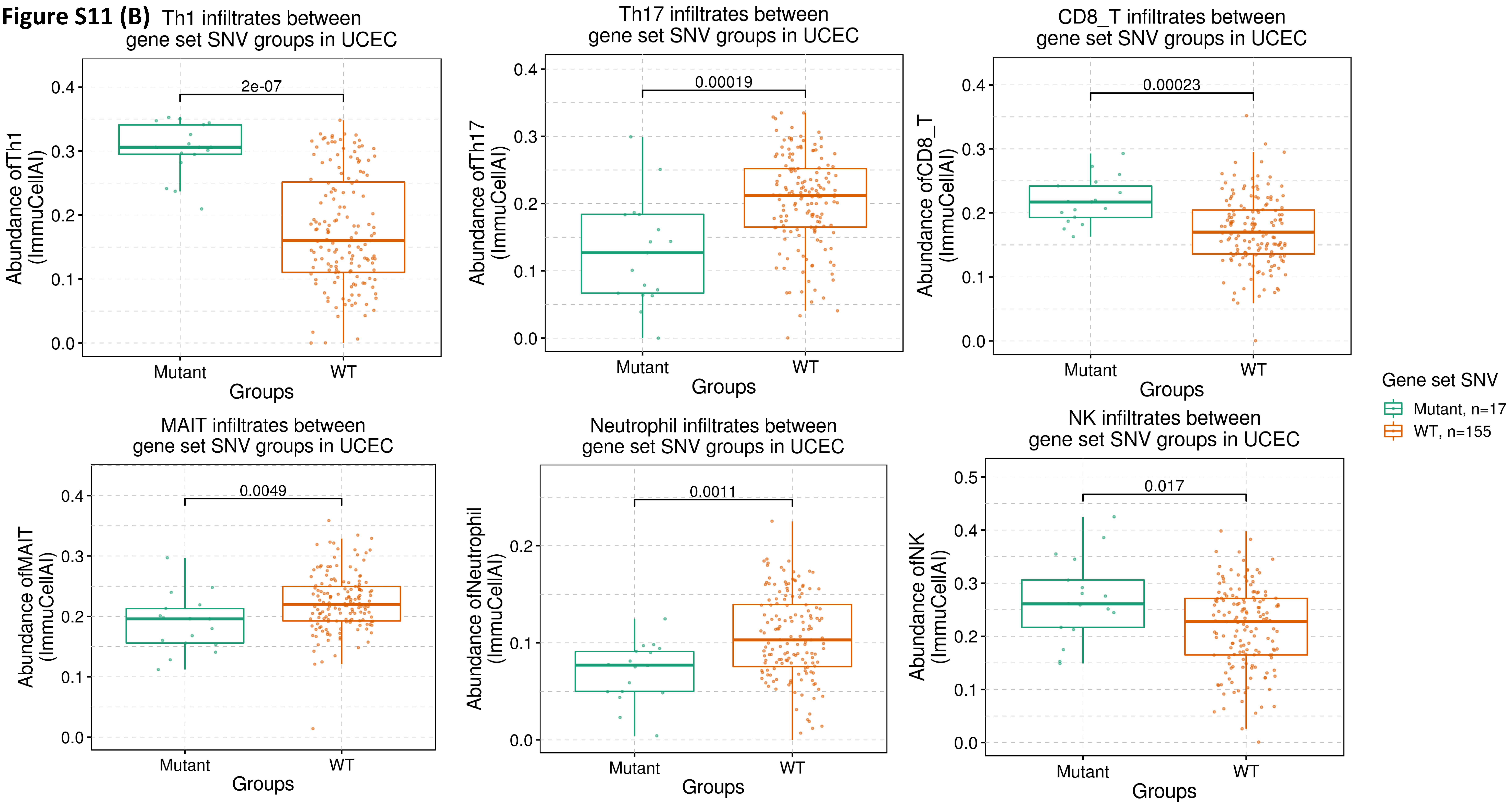
Th1 infiltrates between gene set SNV groups in UCEC
Abundance ofTh1 (ImmuCellAI)
2e-07
0.0
0.1
0.2
0.3
0.4
Mutant
WT
Groups
Th17 infiltrates between gene set SNV groups in UCEC
Abundance ofTh17 (ImmuCellAI)
0.00019
CD8_T infiltrates between gene set SNV groups in UCEC
Abundance ofCD8_T (ImmuCellAI)
0.00023
MAIT infiltrates between gene set SNV groups in UCEC
Abundance ofMAIT (ImmuCellAI)
0.0049
Neutrophil infiltrates between gene set SNV groups in UCEC
Abundance ofNeutrophil (ImmuCellAI)
0.0011
NK infiltrates between gene set SNV groups in UCEC
Abundance ofNK (ImmuCellAI)
0.017
0.5
Gene set SNV
Mutant, n=17
WT, n=155

Figure S11 (C)

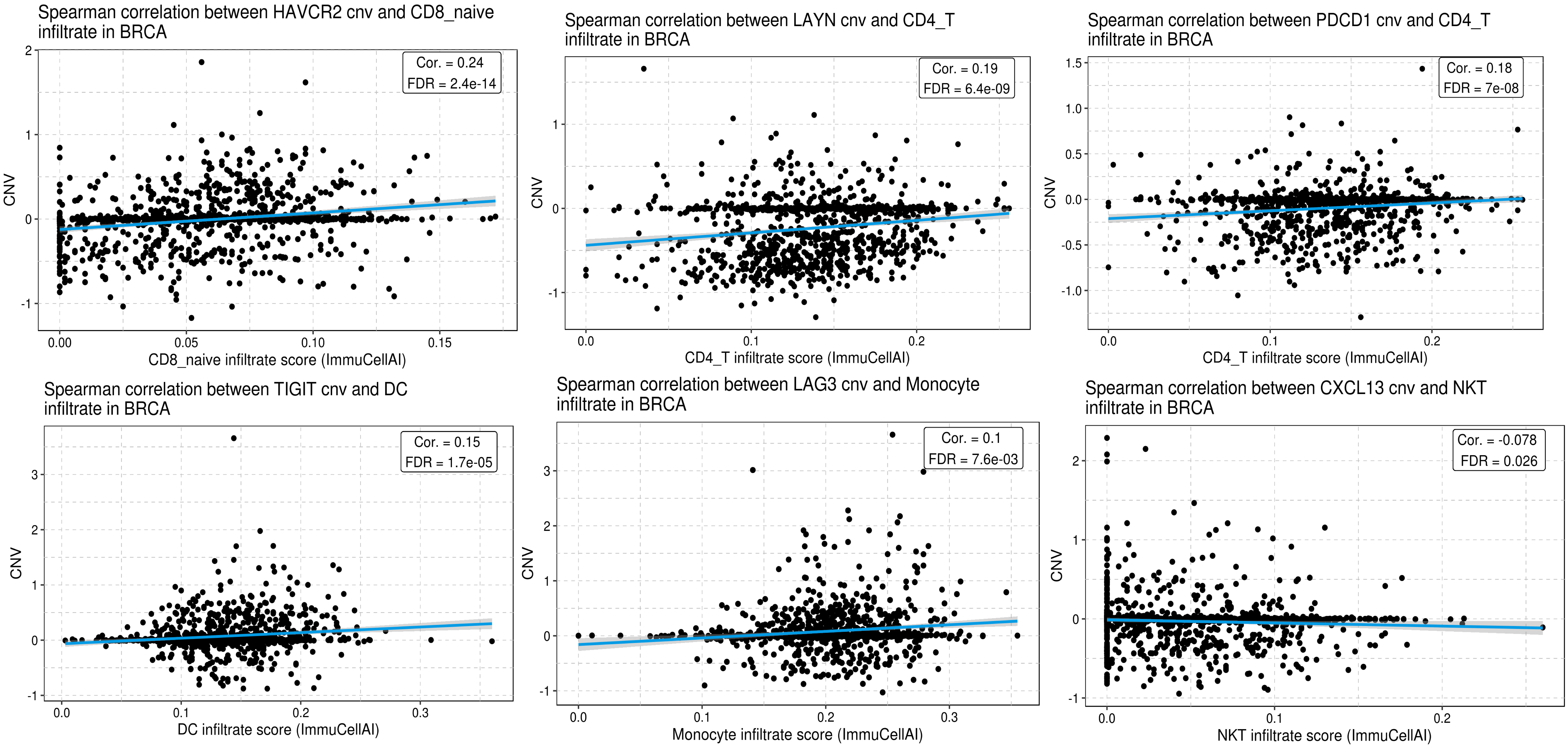
Spearman correlation between HAVCR2 cnv and CD8_naive infiltrate in BRCA
Cor. = 0.24
FDR = 2.4e-14
CNV
CD8_naive infiltrate score (ImmuCellAI)
Spearman correlation between LAYN cnv and CD4_T infiltrate in BRCA
Cor. = 0.19
FDR = 6.4e-09
CNV
CD4_T infiltrate score (ImmuCellAI)
Spearman correlation between PDCD1 cnv and CD4_T infiltrate in BRCA
Cor. = 0.18
FDR = 7e-08
CNV
CD4_T infiltrate score (ImmuCellAI)
Spearman correlation between TIGIT cnv and DC infiltrate in BRCA
Cor. = 0.15
FDR = 1.7e-05
CNV
DC infiltrate score (ImmuCellAI)
Spearman correlation between LAG3 cnv and Monocyte infiltrate in BRCA
Cor. = 0.1
FDR = 7.6e-03
CNV
Monocyte infiltrate score (ImmuCellAI)
Spearman correlation between CXCL13 cnv and NKT infiltrate in BRCA
Cor. = -0.078
FDR = 0.026
CNV
NKT infiltrate score (ImmuCellAI)

Figure S11 (D)

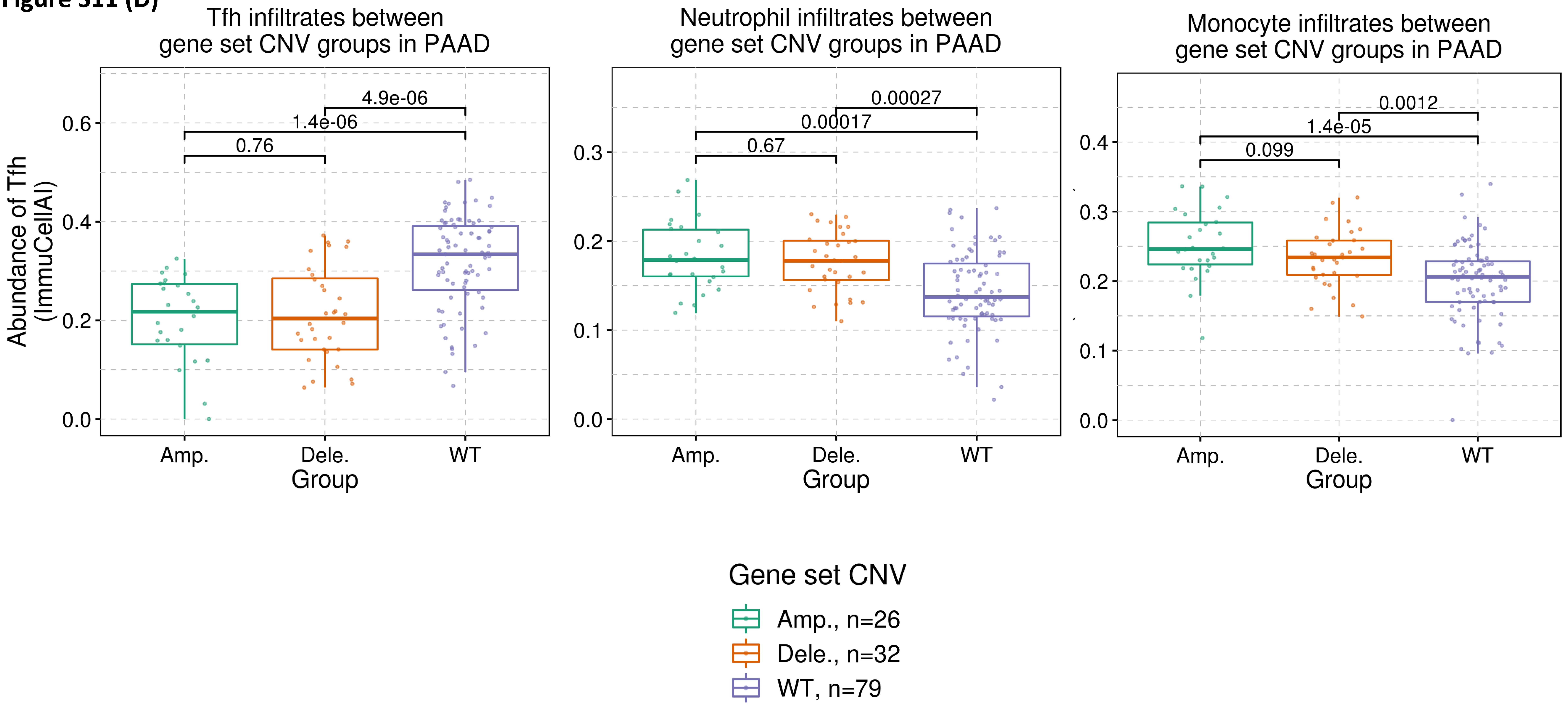
Tfh infiltrates between
gene set CNV groups in PAAD
Abundance of Tfh
(ImmuCellAI)
4.9e-06
1.4e-06
0.76
0.0
0.2
0.4
0.6
Amp.
Dele.
WT
Group
Neutrophil infiltrates between
gene set CNV groups in PAAD
0.00027
0.00017
0.67
0.0
0.1
0.2
0.3
Amp.
Dele.
WT
Group
Monocyte infiltrates between
gene set CNV groups in PAAD
0.0012
1.4e-05
0.099
0.0
0.1
0.2
0.3
0.4
Amp.
Dele.
WT
Group
Gene set CNV
Amp., n=26
Dele., n=32
WT, n=79

Figure S12 (A)

anti-Her2 therapy

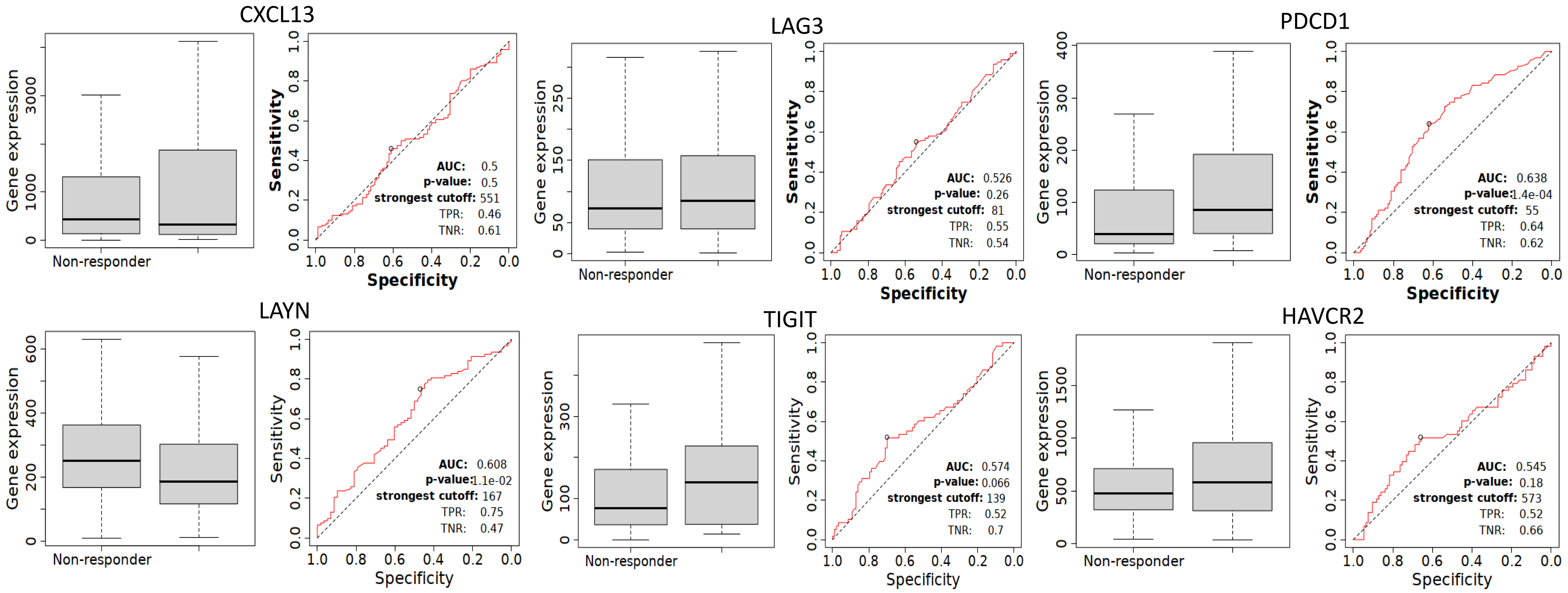

Endocrine Therapy

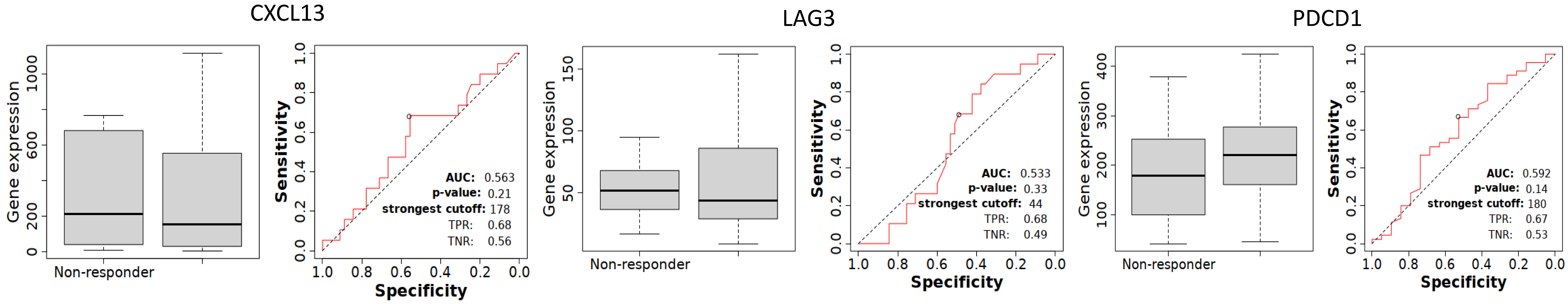

Figure S12 (B) Relapse-free survival at 5 years

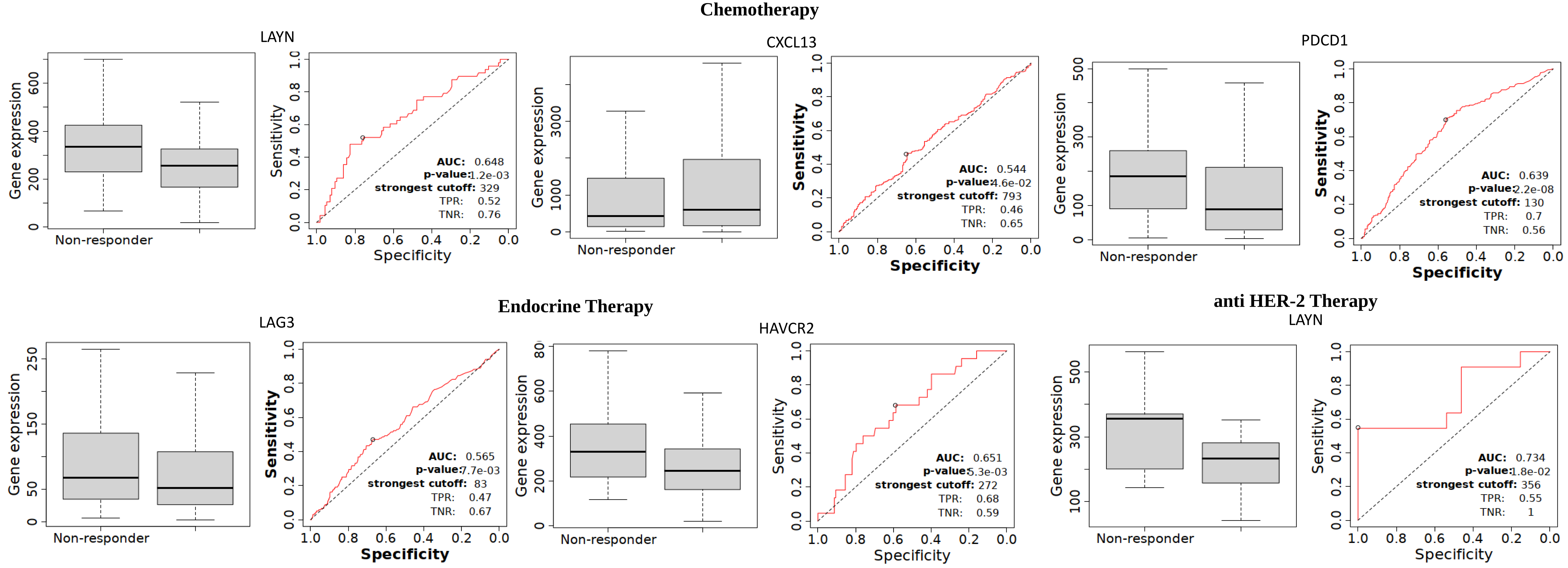

Figure S12 (C)

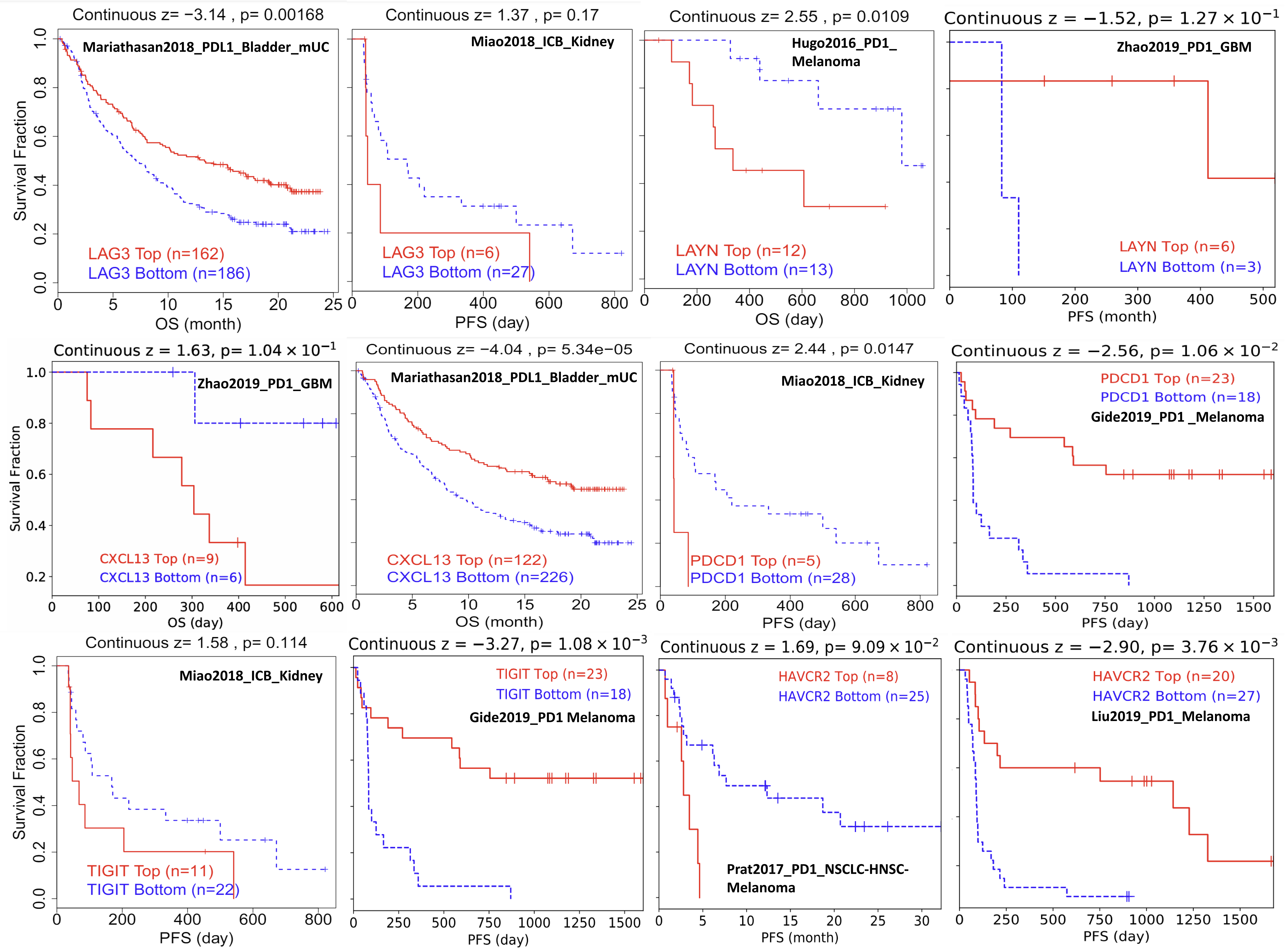

Continuous z= −3.14 , p= 0.00168
Mariathasan2018_PDL1_Bladder_mUC
LAG3 Top (n=162)
LAG3 Bottom (n=186)
Survival Fraction
OS (month)
Continuous z= 1.37 , p= 0.17
Miao2018_ICB_Kidney
LAG3 Top (n=6)
LAG3 Bottom (n=27)
PFS (day)
Continuous z= 2.55 , p= 0.0109
Hugo2016_PD1_ Melanoma
LAYN Top (n=12)
LAYN Bottom (n=13)
OS (day)
Continuous z = −1.52, p= 1.27 × 10^−1
Zhao2019_PD1_GBM
LAYN Top (n=6)
LAYN Bottom (n=3)
PFS (month)
Continuous z = 1.63, p= 1.04 × 10^−1
Zhao2019_PD1_GBM
CXCL13 Top (n=9)
CXCL13 Bottom (n=6)
Survival Fraction
OS (day)
Continuous z= −4.04 , p= 5.34e−05
Mariathasan2018_PDL1_Bladder_mUC
CXCL13 Top (n=122)
CXCL13 Bottom (n=226)
OS (month)
Continuous z= 2.44 , p= 0.0147
Miao2018_ICB_Kidney
PDCD1 Top (n=5)
PDCD1 Bottom (n=28)
PFS (day)
Continuous z = −2.56, p= 1.06 × 10^−2
PDCD1 Top (n=23)
PDCD1 Bottom (n=18)
Gide2019_PD1 _Melanoma
PFS (day)
Continuous z= 1.58 , p= 0.114
Miao2018_ICB_Kidney
TIGIT Top (n=11)
TIGIT Bottom (n=22)
Survival Fraction
PFS (day)
Continuous z = −3.27, p= 1.08 × 10^−3
TIGIT Top (n=23)
TIGIT Bottom (n=18)
Gide2019_PD1 Melanoma
PFS (day)
Continuous z = 1.69, p= 9.09 × 10^−2
HAVCR2 Top (n=8)
HAVCR2 Bottom (n=25)
Prat2017_PD1_NSCLC-HNSC-Melanoma
PFS (month)
Continuous z = −2.90, p= 3.76 × 10^−3
HAVCR2 Top (n=20)
HAVCR2 Bottom (n=27)
Liu2019_PD1_Melanoma
PFS (day)
